# 4D Printing of Humidity‐Driven Seed Inspired Soft Robots

**DOI:** 10.1002/advs.202205146

**Published:** 2023-02-01

**Authors:** Luca Cecchini, Stefano Mariani, Marilena Ronzan, Alessio Mondini, Nicola M. Pugno, Barbara Mazzolai

**Affiliations:** ^1^ Bioinspired Soft Robotics Laboratory Istituto Italiano di Tecnologia Via Morego 30 Genova 16163 Italy; ^2^ Laboratory for Bioinspired Bionic Nano Meta Materials and Mechanics Department of Civil Environmental and Mechanical Engineering University di Trento Via Mesiano 77 Trento 38123 Italy; ^3^ School of Engineering and Materials Science Queen Mary University of London Mile End Road London E1 4NS UK

**Keywords:** 4D printing, biodegradable polymers, bioinspiration, hygroscopic actuation, soft robotics

## Abstract

Geraniaceae seeds represent a role model in soft robotics thanks to their ability to move autonomously across and into the soil driven by humidity changes. The secret behind their mobility and adaptivity is embodied in the hierarchical structures and anatomical features of the biological hygroscopic tissues, geometrically designed to be selectively responsive to environmental humidity. Following a bioinspired approach, the internal structure and biomechanics of *Pelargonium appendiculatum* (L.f.) Willd seeds are investigated to develop a model for the design of a soft robot. The authors exploit the re‐shaping ability of 4D printed materials to fabricate a seed‐like soft robot, according to the natural specifications and model, and using biodegradable and hygroscopic polymers. The robot mimics the movement and performances of the natural seed, reaching a torque value of ≈30 µN m, an extensional force of ≈2.5 mN and it is capable to lift ≈100 times its own weight. Driven by environmental humidity changes, the artificial seed is able to explore a sample soil, adapting its morphology to interact with soil roughness and cracks.

## Graphical abstract

The internal structure and biomechanics of *Pelargonium appendiculatum* (L.f.) wild seeds are investigated to develop a model for the design and 4D printing of a soft robot. The robot mimics the movement and performances of the natural seed. Driven by environmental humidity, the artificial seed can explore a sample soil, adapting its morphology to interact with soil roughness and cracks.

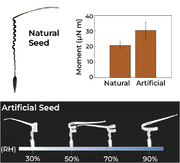

## 1 Introduction

The emerging field of soft robotics has attracted increasing attention for its ability to build shape‐changing intelligent machines, taking advantage of the adaptability and deformability properties of the materials involved.^[^ ^1^, ^2^, ^3^, ^4^, ^5^, ^6^, ^7^, ^8^ ^]^ In environmental applications, a soft robot should be able to move, grow and/or evolve, adapting its morphology to the environmental stimuli and biodegrade at the end of its life cycle.^[^ ^9^, ^10^ ^]^ Furthermore, the increase in energy demand in the field of robotics requires a new class of Embodied Energy autonomous robots,^[^ ^11^ ^]^ which use environment renewable energy to perform their functions with spatial‐temporal continuity.^[^ ^12^ ^]^

In this framework, the bioinspiration for soft robots matches or even exceeds the extraordinary versatility and multifunctionality of natural organisms, such as microorganisms, seeds, plants or animals. The Geraniaceae seeds (e.g., *Erodium* or *Pelargonium* genus’) combine explosive dispersal strategy with a hygroscopic motion to promote germination, providing autonomous motion on terrain surface and penetration into soil fractures. Crawling and burying occur thanks to the hygroscopic seed helical unit (the awn) that responds to variations of external humidity by changing its configuration.^[^ ^13^ ^]^ The metabolically inactive tissues in the awn are responsible for the passive movement of the seed, characterized by a combination of bending and torsional deformation. This occurs thanks to the internal hierarchical structure of the awn, described as a composite material, in which the hygroscopic active element (crystalline cellulose microfibrils) is embedded in a hygroscopic passive soft matrix of structural proteins, polysaccharides and aromatic compounds.^[^ ^14^ ^]^ Cellulose shows hydrophilic moiety, allowing water molecules to store between intermolecular hydroxyl groups via hydrogen bonding.^[^ ^13^, ^14^, ^15^, ^16^, ^17^ ^]^ Therefore, water adsorption determines a volumetric transversal expansion of the cellulose tissues. The volume increase between intermolecular chains or networks leads to the swelling of the wet tissues. Because water adsorption is a reversible process, the swollen tissue of the seed can shrink back to its initial size when dried.

Here we report the first soft robot biomimetic to *Pelargonium appendiculatum* (L.f.) Willd seed in terms of geometry, actuation mechanisms, and biomechanical performances. The robotic seed autonomously explores soil and penetrates inside fractures, extracting energy from the environmental humidity changes.

We performed a morphometric, histological, and biomechanical investigation on the natural seed to predict the kinematic and deformation behaviors of the natural hygroscopic actuator. Then, we exploited the biomechanical specifications to model and design the seed‐like soft robot.

The development of such autonomous soft robots required the use of advanced technology for the fabrication. Considering emerging additive manufacturing techniques, 4D printing permits to replicate natural physio‐mechanical variations over time.^[^ ^18^, ^19^ ^]^ 4D printed structures have the unique ability to create dynamic morphological changes under environmental stimuli (i.e., humidity), generating environmental propulsion. According to the material design, structures can be programmed to re‐shape and to perform work, adapting its morphology to the selected scenario.

We used 4D printing techniques with biodegradable polymers for the realization of the artificial seed, including Fused Deposition Modeling (FDM) of polycaprolactone (PCL, hygroscopic inactive material),^[^ ^20^ ^]^ coupled with coaxial electrospinning of hygroscopically active fibers, composed by a polyethylene oxide (PEO) shell^[^ ^21^ ^]^ and a cellulose nanocrystals (CNC) core.^[^ ^22^ ^]^ The artificial and natural seeds have comparable geometrical dimensions and biomechanical performances. In the natural seed, the measured torque and extensional force were 20.7 ± 2.5 µN m and 5.1 ± 1.1 mN, respectively, while in the artificial one 30.4 ± 5.1 µN m and 2.4 ± 0.6 mN, respectively. Moreover, the developed theoretical model predicts, within the confidence interval, the kinematics and statics of the seeds, making it a tool to design a new class of soft robots that explore topsoil by adapting their morphology according to soil composition, roughness and stiffness.

## 2 Results

### 2.1 Morphology and Biomechanical Analysis of *Pelargonium appendiculatum* for Soft Robot Design

**Figure 1:**
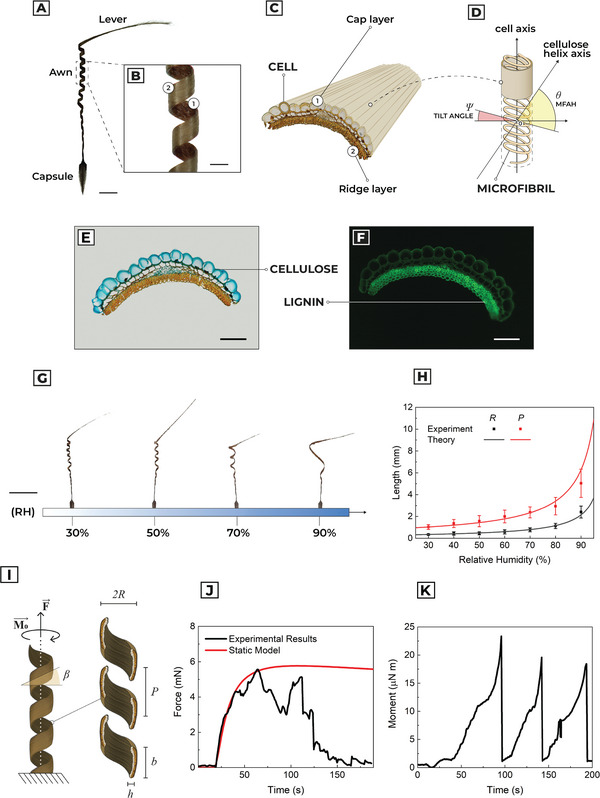
Morphological, compositional and biomechanical characterization of the awn in *P. appendiculatum*. a) Optical image of *P. appendiculatum*. Scale bar: 5 mm. b) Optical magnification of the awn. Scale bar: 500 µm. c) Scheme and nomenclature of the awn. d) Cellulose microfibril distribution and angle of definition in cap layer cell. e) Transverse section of the awn with cellulose stained with Alcian blue. Scale bar: 100 µm. f) Fluorescent image of a transverse section of the awn with lignin auto‐fluorescence, excitation at 488 nm. Scale bar: 100 µm. g) Experimental visualization of pitch (P) and radius (R) as a function of the relative humidity. The corresponding variations of P and R is shown in Video S1, Supporting Information. Scale bar: 1 cm. h) Comparison between laminate composite model and experimental results in measuring kinematic parameters (pitch and radius) as a function of RH. i) Schematic representation of geometrical parameters involved in the static evaluation of extensional force F and bending plus torsional moment *M*_0_. j) Comparison between time evolution of the extensional force F and relative behavior of the static model. k) Time evolution of the moment *M*_0_.

**Figure** **1** shows the morphological and compositional analysis of a *P. appendiculatum* seed where three sections can be distinguished (Figure 1a): i) the capsule, containing the seed embryo, which anchors the structure into the soil and converts rotational motion into burial behavior through anisotropic friction;^[^ ^14^ ^]^ ii) the awn (Figure 1b), which acts as an independent hygroscopic actuator, thanks to the helical arrangement of cellulose microfibrils along the cylindrical cells of the cap layer^[^ ^14^, ^15^, ^16^, ^17^ ^]^ (Figure 1c,d); and iii) the lever, which functions as a passive element that allows the self‐lifting of the seed and applies torque acting as an anchoring element.^[^ ^14^, ^15^, ^16^, ^17^ ^]^

Section S1 and Figure S1, Supporting Information, report a detailed morphometric analysis of a Pelargonium awn and lever structures. Transverse sections of the awn show a bilayered (Figure 1e,f) string‐like shape composed of lignin‐rich sclerenchyma cells and a layer of larger cells aligned in parallel with each other. When coiling, the sclerenchyma is the outer layer of the coil (ridge layer), and the large cell tissue is the inner one (cap layer). Alcian blue histochemical stain shows the asymmetric localization of cellulose in the internal tissue of the awn (Figure 1e), while lignin distribution was confirmed in all tissue (Figure 1f), especially sclerenchyma, by its auto‐fluorescence with blue light excitation (488 nm). In this frame, the ridge layer enacts the role of a hydrophobic passive substrate that prevents delamination of cellulose due to water solubilization. Relative thicknesses evaluated through SEM, bright and dark field microscopy of the section, show that the thickness of the cap and ridge layer are, respectively, *h*_Cap_= 39.8 ± 6.9 µm and *h*_Ridge_= 59.7 ± 9.8 µm (n = 6).

In the lever region (Figures S2 and S3, Supporting Information), the sections lose their string shape, for a rounder one, with the inner tissue having a smaller cell size and the sclerenchyma tissue more extension in comparison to the awn. Since the concentration of cellulose is uniformly distributed (Figure S2c,d, Supporting Information), the lever is a non‐hygroscopic element of the structure.

The main structural difference between the *P. appendiculatum* awn with other Geraniaceae seeds, for example, *Erodium* genus, is the morphological distribution of cellulose (Figure S4, Supporting Information). The *Erodium* awn is characterized by homogeneous‐sized cells in the inner tissues with a gradient cellulose concentration along its thickness.^[^ ^6^, ^15^ ^]^

Due to its compositional and morphological nature, the awn in *P. appendiculatum* can be split into an effective bilayer, which makes it more suitable for the design and bioinspiration of an artificial equivalent seed based on bilayered hygroscopic structure.^[^ ^23^ ^]^

The cap and ridge layer stiffness were analyzed separately, due to different morphological and compositional structures. Considering the small volumes of material involved, Young's modulus cap and ridge layer are characterized by the nanoindentation technique at fixed humidity and temperature (RH = 50%, *T* = 25 °C): *E*_Cap_ = 0.78 ± 0.29 GPa and *E*_Ridge_ = 1.48 ± 0.25 GPa (*n* = 5). From a biomechanical point of view, the dried tissue in Pelargonium mediates the hygroscopic movement and it is composed of a combination of lignin and hemicellulose, as in the pinecone. So, the linear coefficient of hygroscopic expansion (CHE) of the cap and ridge layer is selected according to Dawson et al.^[^ ^24^ ^]^ measurement: *α*_Cap_ = 0.20 ± 0.04 and *α*_Ridge_ = 0.06 ± 0.02.

The coiling configuration of the awn is determined by the geometrical arrangement of the microfibrils that causes an anisotropic hygroscopic expansion along the main direction, defined by the microfibril angle (MFA). Since this angle is tilted compared to the cell axis, it is possible to define two angles to describe microfibril expansion: the tilt angle *Ψ*, which is the angle between the cellulose helix axis and the cell axis, and the cellulose microfibril angle *θ* in relation to the cellulose helix axis (MFAH) (Figure 1d). Tilt angle and MFAH are already reported and measured by using small‐angle X‐ray scattering (SAXS):^[^ ^15^, ^25^ ^]^ *Pelargonium peltatum* and *appendiculatum* show a tilt angle of 15° ± 5° and MFAH ranging from 70° to 80°. Furthermore, from a macroscopic point of view, the helical motion of the awn can be predicted considering the radius (*R*), which represents half of the cylindrical helix diameter, and the pitch (*P*), or rather the spatial period of the windings along the cylinder helix axis, as a function of RH *ϕ*.

To predict the kinematic behavior of *P. appendiculatum*, Ha et al.^[^ ^25^ ^]^ proposed to separate the hygroscopic structure into a trilayer (Figure S5, Supporting Information). In this way, it is possible to model the kinematic deformation of the awn using the theory of laminate composite anisotropic plates, where only thickness is involved in the deformation. Considering that the stress generated in the structure is only dependent on hygroscopic swelling, the whole stress can be modeled accordin*g* *to* *σ* = *D* *α*Δ*ϕ* where *σ* is the stress, *D* is material stiffness, *α* is CHE and Δ*ϕ* is the variation of RH in relation to humidity saturation concentration (see Section S2, Supporting Information).

Figure 1g and Video S1, Supporting Information, show pitch and radius variation as a function of *ϕ* measured in a climatic chamber at 30 °C, with humidity increase from 30% to 90% with 10% step variations in Δ*ϕ* (Figure S6, Supporting Information). Each level of humidity was fixed for at least 5 min, to overcome the moisture diffusion limit in the cap layer. Figure 1h shows that kinematic modeling predicts coherently the geometrical variation of P and R in the whole range of RH considered.

The ability of the seed to move, interact with objects, self‐dig and consequently explore soil is mediated by the extensional force *F* and moment *M*_0_ (combination of bending and torsion) that can be generated by the awn during the variation of %RH (Figure 1i). It is possible to model such static parameters considering the elastic theory: we consider the awn as a homogeneous open‐coiled cylindrical helical spring with a rectangular cross‐section subjected to large deflections.^[^ ^26^ ^]^ Moreover, water permeation in the cap layer tissues is modeled considering 1D Fickian diffusion along the thickness axis (*z*‐axis), where a constant concentration source *ϕ*_0_ is located at the top of the surface (*z* = 0).

So, *F* and *M*_0_ can be estimated in a closed‐form solution:^[^ ^26^ ^]^

(1) $$\begin{array}{ccc} F\;\left(\phi \right) & = & \frac{Gk_{s}bh^{3}\cos\beta }{R\left(\phi \right)}\left(\frac{\sin\beta \cos\beta }{R\left(\phi \right)}-\frac{\sin\beta_{0}\cos\beta_{0}}{R_{0}}\right)\\ & & -\;\frac{E\;bh^{3}\sin\beta }{12\;R\left(\phi \right)}\left(\frac{\cos^{2}\beta }{R\left(\phi \right)}-\frac{\cos^{2}\beta_{0}}{R_{0}}\right) \end{array}$$

(2) $$\begin{array}{ccc} M_{0}\;\left(\phi \right) & = & Gk_{s}bh^{3}\sin\beta \left(\frac{\sin\beta \cos\beta }{R\left(\phi \right)}-\frac{\sin\beta_{0}\cos\beta_{0}}{R_{0}}\right)\\ & & +\;\frac{Ebh^{3}\cos\beta }{12}\left(\frac{\cos^{2}\beta }{R\left(\phi \right)}-\frac{\cos^{2}\beta_{0}}{R_{0}}\right) \end{array}$$

(3) $$\phi \;\left(z,t\right)=\Delta \phi_{s}\;\mathrm{erfc}\left(\frac{z}{2\sqrt{\mathrm{Dt}}}\right)+\phi_{0}$$

where *F* is extensional force, *G* the shear modulus, *k*_s_ the shape factor, *b* the width, *h* the thickness, *β* the coiling angle, *β*_0_ the coiling angle at the initial RH state, *R*_0_ the radius at the initial RH state, *E* the Young's modulus, *I* the moment of inertia of rectangular cross section, *t* the time, *z* the thickness direction, *ϕ*_0_ the RH concentration at *ϕ*(h, 0), Δ*ϕ*_s_ the variation of saturation concentration with respect to *ϕ*_0_, and $\sqrt{Dt}$ the diffusion length.

Since water diffusion in the cap layer is mediated by Fickian mechanism,^[^ ^14^, ^15^, ^16^, ^17^, ^25^, ^27^ ^]^ we first provide an evaluation of diffusivity *D*, defined as $D=h_{c}^{2}/t_{s}$, where *h*_c_ is the thickness of cap layer and *t*_s_ is diffusion time: 2.3 ± 0.7 × 10^−11^ m^2^ s^−1^.

We experimentally measured the torque at the end of the lever (which represents the moment arm) and the extensional force during uncoiling (Figure S7, Supporting Information), by abruptly increasing RH from 35% to 90% using water aerosol (Figure S8b, Supporting Information). In Figure 1j, we report a comparison between the experimental and modeling results of the extensional force: considering well‐fitted prediction of static behavior, time‐variation in experimental results is mainly associated with buckling. Maximum force is reached at *t* = 65 ± 12 s, with a value of *F*_Max_ = 5.1 ± 1.1 mN, while the model prediction is *F*_Model_ = 5.6 mN. The reduction of extensional force is not only associated with Young's modulus reduction due to increasing water content in the cap layer tissue,^[^ ^14^ ^]^ but it is also related to the reduction of spring deflection with the increase of RH.

Figure 1k shows the moment measured during the uncoiling rotation. The maximum moment modulus is equal to 20.7 ± 2.5 µN m reached at time 104 ± 23 s, while the model prediction is 57.7 *µ*N m. This overestimation (within the same order of magnitude) is because the model does not consider the buckling effect: abrupt variation of moment is repeated cyclically because the awn periodically loses contact with the force sensor due to cylindrical axis deformation.

Considering that the model predicts within the confidence interval kinematic and static of awn of *P. appendiculatum*, we used it as a reference calibration to design the hygroscopic actuator for the artificial seed.

### 2.2 4D Printing of the Hygroscopic Actuator

4D printed structures can reshape in a pre‐programmed and passive manner over time due to RH changes and may be accompanied with function evolvement in the process.^[^ ^18^, ^19^ ^]^ We exploited this advantage to realize a hygroscopic actuator capable of mimicking the deformation of the awn through a bilayer structure. The inactive layer was fabricated through FDM printing of PCL thermoplastic. Then, we activate the hydrophobic PCL surface with oxygen plasma to increase the adhesion with hydrophilic fibers. Finally, we deposited on the top of the substrate PEO/CNC hygroscopic fibers using coaxial electrospinning.^[^ ^28^ ^]^ The coupling of FDM and electrospinning techniques permits to realize a support inactive structure independently from the design of active fibers, increasing the degrees of freedom in the prototyping of soft robots.

**Figure 2:**
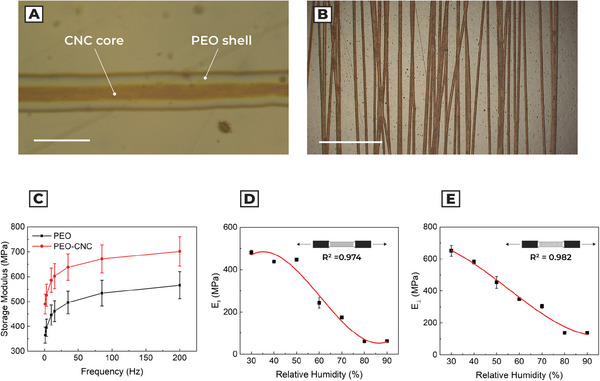
Characterization of the fibrous active layer. a) Optical image of a coaxial fiber. The shell is composed of PEO and the core by CNC. The difference in color of the fibers is due to the birefringence caused by the different refractive indexes of the involved materials. Scale bar: 15 µm. b) One round trip cycle of electrospun fibers on the sample substrate. Scale bar: 500 µm. c) Comparison between storage modulus of PEO fibers and coaxial PEO/CNC fibers through dynamic nanoindentation. d) Fibers longitudinal Young's modulus as a function of the relative humidity. e) Fibers transversal Young's modulus as a function of the relative humidity.

Electrospun hygroscopic fibers compose the active layer, formed by a core element of CNC and a shell of PEO (**Figure** **2**a). The two materials are characterized by an enzymatic biodegradable behavior.^[^ ^21^, ^22^ ^]^ The coaxial electrospinning approach has two main advantages in developing hygroscopic actuators.^[^ ^29^ ^]^ First, CNC shows a reinforced stiffness potential when included in a polymer matrix, increasing the hygroscopic deformation capabilities.^[^ ^30^, ^31^ ^]^ Second, considering that water adsorption is a superficial phenomenon, the PEO CHE is not altered by CNC since they are fully included in the PEO shell.

In Figure 2b, and Figures S9 and S10, Supporting Information, we evaluate the morphology and orientation of fibers. The mean diameter of a fiber is 12.7 ± 0.9 µm (*n* = 33), with a CNC core diameter of 5.4 ± 1.2 µm (*n* = 10). Furthermore, the fibers show an orientation degree of 90° ± 3°, therefore the fibers mainly align along the electrospun direction.

We performed dynamic nanoindentation on the polymeric fibers (Figure 2c and Figure S11, Supporting Information). The obtained results show a statistically relevant increase of Storage modulus compared to homogeneous PEO fibers (paired *t*‐test p* < 0.05), increasing the value by 34.62%, from 364.5 ± 31.9 to 490.2 ± 39.8 MPa, with constant temperature *T* = 25 °C and humidity RH = 50%. To understand the mechanical behavior of the bilayers, we carried out a tensile test in a climatic chamber to define Young's modulus of the PEO/CNC fibers layer (Figure 2d,e and Figure S12, Supporting Information). The material shows a decay in stiffness with the increase of RH, both in longitudinal and transversal arrangements. Experimental results are interpolated by cubic‐polynomial fitting. Although the mechanical properties of the fiber layer are anisotropic, the addition of the CNC core increases the transversal stiffness compared to the experimental results on PEO fibers provided in the literature.^[^ ^32^ ^]^ Since the hygroscopic expansion phenomenon involves surface adsorption of water, we consider the CHE value of PEO reported in the literature:^[^ ^25^, ^32^ ^]^ *α*_PEO_ = 0.10 ± 0.03.

The passive layer of the hygroscopic actuator is realized in PCL, a thermoplastic semi‐crystalline aliphatic polyester. This material is selected because of its biodegradability, both hydrolytic and enzymatic,^[^ ^20^ ^]^ chemical resistance, humidity inertness, flexibility and elongation capabilities.^[^ ^20^ ^]^ Furthermore, it is suitable to print with FDM in a one‐step process and apply geometrical features according to a specific design.

We printed testing beams in PCL with length *l* = 30 ± 0.5 mm, width *w* = 5.0 ± 0.5 mm and thickness *t* = 123.3 ± 6.8 µm (*n* = 5). Considering the low melting point of PCL (≈60 °C), we chose to print on a heated plate (40 °C) to fuse the patterned fibers. The result is a homogeneous flat substrate with no preferential direction (isotropic behavior). To increase adhesion between hydrophobic PCL and water‐based electrospun fibers, we performed a surface activation through oxygen plasma, and we verified the effects of the treatment through contact angle measurement: samples processed with 150 W plasma for 30 s show a statistically significant (paired *t*‐test p* < 0.05) reduction of the contact angle from 69.8° ± 1.1° to 26.6° ± 3.3° (Figure S13, Supporting Information). Moreover, to understand the effects of plasma treatment on elastic modulus, we carried out dynamic nanoindentation on PCL structure. This characterization shows that treatment does not change with a statistical relevance the Storage Modulus (paired *t*‐test p* > 0.05): 254.3 ± 16.1 MPa at a testing frequency of 1 Hz (*n* = 5) (Figure S14, Supporting Information).

**Figure 3:**
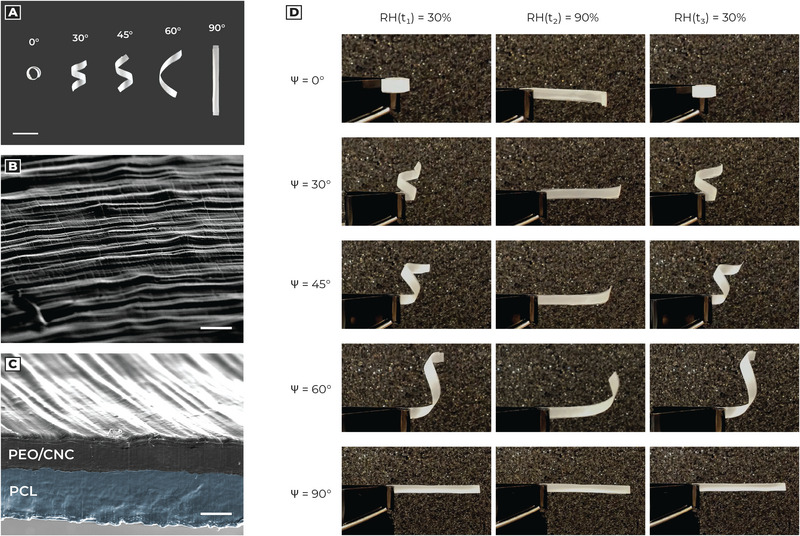
Deformation and morphology of bilayered beams. a) Deformation behavior of bilayered beams, with fibers oriented at different angles. b) SEM picture of the beam surface highlighted the fibrous composition at the top of the active layer. Scale bar: 50 µm. c) SEM picture of the beam cross‐section, realized through blade cut. The sample preparation for this analysis required a dehydration protocol, in which the hygroscopic actuator was heated at 40 °C under vacuum overnight. Then, we proceeded to cut the structure with a lancet for the cross‐section visualization. Because of these destructive operations, the fibers tend to conglomerate, avoiding the observation of the internal structure. Scale bar: 40 µm. d) Reversible behavior of bilayered beams at different RH as a function of fiber deposition angle.

We then manufactured five flat bilayered beams (**Figure** **3**a and Figure S15, Supporting Information) with electrospinning, depositing fibers with different orientations (0°, 30°, 45°, 60°, 90°).

In Figures 3b and 3c we can observe SEM images of 90° sample, both in surface and cross‐section. Fibers tend to conglomerate in a continuous structure during deposition, maintaining their directionality. From a morphological point of view, we deposited an active layer of thickness equal to *h_a_* = 72.9 ± 6.5 µm.

Processed structures show bending and torsional deformation thanks to the fiber tilt angle and the relative longitudinal expansion of hygroscopic fibers. Specimens show reversible coiling/uncoiling behavior as the awn in Pelargonium (Figure 3d and Video S2, Supporting Information).

### 2.3 Design and Performance of the Artificial Seeds

We designed a seed‐like soft robot, following a biomimetic approach, based on biomechanical characterization of *P. appendiculatum*, the modeling procedure, as well as the 4D printing technique and materials characterization previously reported. The whole design, 4D printing process, and material training are reported in Figures S16–19, Supporting Information.

The design of the artificial capsule was processed considering the contour of the natural one and adapting the relative dimension, which maintains the same scale factor as to the whole size of the artificial seed (Figure S17, Supporting Information). The artificial lever is designed considering a triangular profile with a length *r* = 17 ± 1 mm (*n* = 5).

**Figure 4:**
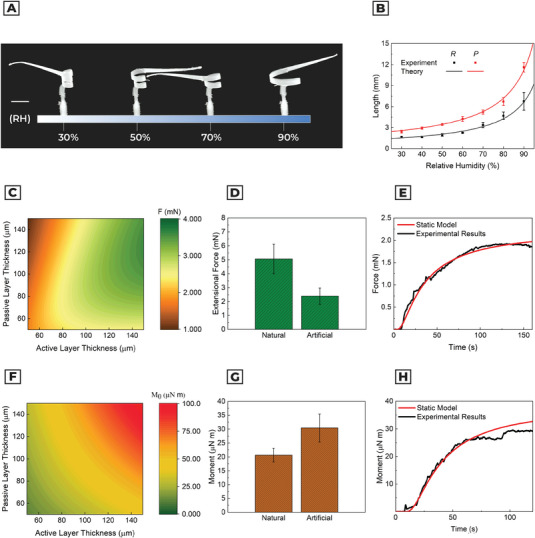
Kinematic and static characterization of the artificial seed. a) Experimental visualization of pitch and radius as a function of relative humidity. The corresponding variation of P and R is shown in Video S3, Supporting Information. b) Comparison between laminate composite model and experimental results in measuring kinematic parameters (pitch and radius) as a function of RH. c) Modeling results of the extensional force for the artificial seed as a function of passive and active layer thickness, considering width *B* = 1523 µm, length *L* = 30 mm, tilt angle 15° and printing direction 70°. d) Comparison between natural and artificial seed performances in extensional force, considering width *B* = 1523 µm, length *L* = 30 mm, tilt angle 15°, and printing direction 70°. e) Extensional force comparison between experimental and modeling results. f) Modeling results of the moment for the artificial seed, as a function of passive and active layer thickness. g) Comparison between the experimental evaluation of moment in the natural and artificial seeds. h) Moment comparison between experimental and modeling results.

In agreement with the model, we selected width *b* = 1523 ± 54 µm, length *l* = 30.3 ± 1.1 mm, passive layer thickness of *h*_p_ = 100.7 ± 4.6 µm, and active layer thickness *h*_a_ = 72.9 ± 6.5 µm (*n* = 5) (Figure S20, Supporting Information). Following a biomimetic approach, we set the direction of the fibers deposition equal to the tilt angle Ψ = 15° of the natural seed, to better compare natural and artificial seed performances. We recall that PEO/CNC fibers present a longitudinal hygroscopic expansion, while pure cellulose is subjected to transversal expansion.^[^ ^16^, ^17^, ^18^, ^25^ ^]^ After fiber deposition, the artificial awn starts to deform according to the humidity level. We then accelerated the deformation transient process (i.e., material training) by changing the humidity level in the climatic chamber. Consequently, they show a completely reversible and reproducible (*n* = 15) behavior after 5 ± 1 humidity cycles (from RH = 30% to 90%). Overall, the volumetric dimension of the artificial seeds is 10 times bigger than the natural ones (Figure S21, Supporting Information). The mass of the device is equal to 21.6 ± 0.4 g (four times the mass of a natural seed), while the artificial awn shows a mass of 12.6 ± 0.6 g (*n* = 5). Next, we evaluated the equivalent kinematic parameters of the artificial seed following the same characterization procedures used for *P. appendiculatum*. In **Figure** **4**a,b and Video S3, Supporting Information, we observe the experimental and modeling variation of pitch and radius as a function of RH.

Considering the kinematic variation of the structure, we measured the diffusion constant on the fiber layer, obtaining a diffusivity of 4.7 ± 0.5 × 10^−11^ m^2^s^−1^, almost two times higher than the natural one.

Finally, we evaluated the extensional force (Figure 4c–e and Figure S22a, Supporting Information) and moment (Figure 4f–h and Figure S22b, Supporting Information) in the artificial seed, and performances compared to the natural one. The maximum force is reached at *t* = 123 ± 32 s, with a value of *F*_Max_ = 2.40 ± 0.58 mN, while the model prediction is *F*_Model_ = 2.0 mN. Moment measurement exhibited a maximum value at *t* = 112 ± 24 s, with $M_{0_{\mathrm{Max}}}=30.4\pm 5.1$ µN m and $M_{0_{\mathrm{Model}}}=33.4$ µN m. In this case, the awn does not lose contact with the force sensor, because the structure did not show cylindrical axis deformation. The artificial seed has slower dynamics than *P. appendiculatum* since the law of diffusivity scales linearly with the thickness of the active layer and it is inversely proportional to square root diffusivity (Equation 3).

Upon exposing the artificial seed to 100 cycles of RH variation between 0.3 to 0.9, we found that the amount of change in curvature was less than 10%.

**Figure 5:**
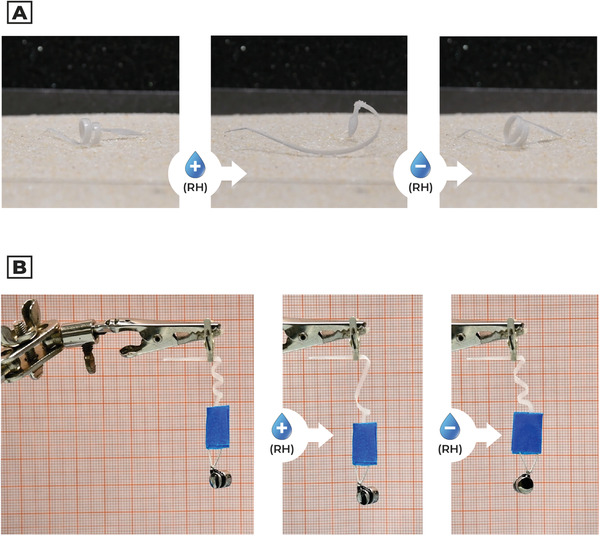
Lifting performances of the artificial seed‐like robot. a) Example of the artificial seed self‐lifting (Video S4, Supporting Information). b) From idle conditions (RH = 35%) the artificial seed is subjected to abrupt variation of humidity with water aerosol (RH = 90%). Water desorption is then promoted by environmental humidity. The corresponding video is shown in Video S5, Supporting Information.

To understand the abilities of the artificial seed in humidity‐driven soil exploration, we first performed a lifting analysis (**Figure** **5**a and Video S4, Supporting Information). Once we observed self‐lifting in artificial samples, we tested the maximum stress performances of the device. We fixed the artificial lever and tied 1 g weight to the artificial capsule and, by changing the humidity level from 35% to 90%, the hygroscopic actuator lifted about 120 times its weight by 5 mm (Figure 5b and Video S5, Supporting Information). Considering that the water adsorption enthalpy is the only source of energy, the absolute potential energy related to the lifting process was ≈ 49 *µ*J. The main limitation associated with this observation is the reduction of ultimate tensile stress of the hygroscopic layer with the increase of RH. Consequently, the fibers have a mechanical yield, which does not allow the structure to return to its initial position.

We investigated the movement of the artificial seed in sample soil: the test consists of monitoring and tracking the spontaneous movements of the seed‐like robot due to humidity changes in randomly distributed clay. The hygroscopic actuator (artificial awn) was used as a biomimetic propulsor, matched with the capsule, and the lever which act as anchoring points (or interaction points) for the hygroscopic actuator. The substrate was composed of variable roughness and fractures to have the widest possible range of dynamic interactions between the terrain and the device. The fractures represent 8.95% of the whole surface, with an average roughness of ≈5 mm. The test was done in a climatic chamber, with *T* = 30 °C and RH changing linearly from 30% to 90% (triangular shaped function). The climatic chamber provides a homogeneous distribution of humidity, so there is not a preferential direction in the RH source. In Video S6, Supporting Information, it is possible to observe the dynamics of four different artificial seeds after 19 humidity cycles. The RH cycle is represented by a triangular waveform *ϕ*(t) with a rise/fall time of 30mins per cycle. In the first approximation, the time‐varying behavior of the specimen can be modeled as a succession of random steps in a 3D continuous space (random walk). Therefore, the initial position of the seed determines the random evolution of the whole system, since the device chaotically interacts with soil and fractures. The reversible shape commutation mechanism is then a successful approach for soil exploration if the soil presents variable geometrical and physical properties since the soft robot progressively moves toward a fracture only if it can interact with the soil roughness.

**Figure 6:**
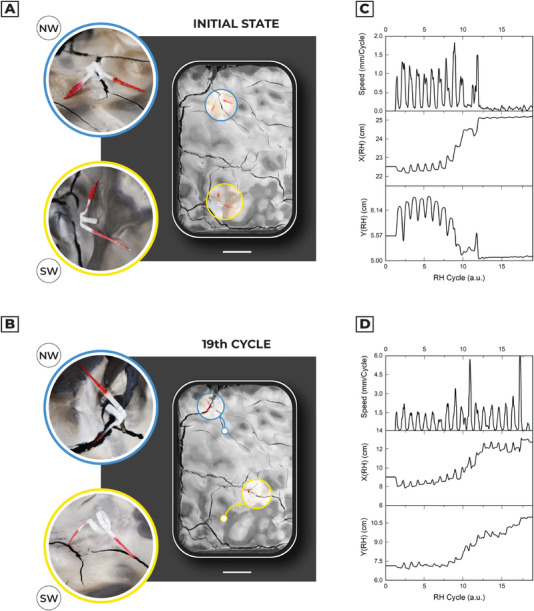
Artificial seeds in humidity‐driven soil exploration. a) The initial condition of two different samples placed on artificial clay soil. The central image represents the top view of the soil. The corner arrangement was chosen to minimize the interaction among the different specimens. Scale bar: 5 cm. b) The final position of the samples after 19 humidity cycles in a climatic chamber (*T* = 30 °C and RH range from 30% to 90%). Due to humidity‐driven motion, the artificial seeds interact with multifaceted soil and its cracks. The evolution of seed movements due to humidity cycles is shown in Video S6, Supporting Information. Scale bar: 5 cm. c) Relative position and speed of the North–West artificial seed obtained by video tracking. d) South–West artificial seeds relative position and speed.

In Figure S23, Supporting Information, we reported the time evolution of the relative position on XY plane and the absolute speed. **Figure** **6** and Figure S24, Supporting Information, summarize the final and initial position of each seed. The coiling/uncoiling behavior is confirmed by motion tracking of the seed capsule since the mean speed measurement follows linearly the duty cycle of periodic humidity input. Moreover, we verified that the dispersing movement of the artificial seeds is mediated by the autonomous adaptation of the soft body across the soil, highlighting different classes of behavior: north–west seed penetrates with the capsule a fracture 3 cm far after 12 humidity cycles (0.63 ± 0.42 mm/cycle); south–west seed presents a directional movement, with a mean speed of 0.93 ± 0.90 mm/cycle, reaching a peak of ≈6 mm/cycle and at the 19th RH cycle anchors both capsule and lever in a fracture; north–east and south–east seeds roll over a confined position during all the experiment, due to lever anchoring with soil fractures.

## 3 Conclusion

In this study, we report the 4D printing of seed‐like soft robots inspired by the hygroscopic dispersion mechanism of Geraniaceae seeds, which can self‐lift and explore soil in response to changes in environmental humidity. We provided a characterization procedure and a theoretical model to predict the force and moment generated by the natural hygroscopic actuator. We exploited the model for the design of the soft robots, which show morphology, actuation mechanism and static performances comparable to *P. appendiculatum*. In Table S1, Supporting Information, we summarize the literature and experimental data used for modeling and design. Among different actuation mechanisms, hygroscopic actuators represent a renewable energy solution for small‐scale robotics with Embodied Energy.^[^ ^9^ ^]^ The natural and artificial hygroscopic seed actuators show an energy density of 564.80 and 52.16 kJ m^−3^ (power‐to‐mass ratio of 423.62 and 154.38 µW kg^−1^), respectively. This makes the hygroscopic actuation suitable for environmental soil exploration.

The experimental energy density of the actuator reaches a peak of ≈ 4 J kg^−1^, value that is comparable with the current state‐of‐art for soft hygroscopic actuators (from 0.5 to 10 J kg^−1^).^[^ ^33^, ^34^, ^35^ ^]^

The ability of the robot to move, explore and adapt its morphology to the soil in specific environmental conditions represents a milestone for the design of artificial multi‐functional materials and soft miniaturized robots with morphological computation behaviors. Considering our findings, there is a trade‐off in hygroscopic actuation, because the increase of geometrical dimensions of seed‐robots will lead to an increase in force and moment, but the dynamics will be slower. Hence, geometrical dimensions, materials choice, fiber directionality must be weighted according to the specific scenario in which the robot is involved. Moreover, the role of biodegradable materials and eco‐friendly processing used in this work, becomes fundamental for sustainable and green robotics to avoid the dispersal of new waste in natural environments and no need for retrieval actions.

In this perspective, the reported seed‐like soft robots could endow sensing abilities and be used as battery‐free wireless tool for environmental top‐soil monitoring,^[^ ^36^, ^37^ ^]^ integrating in the same device sampling and sensing. This could be a simple and low‐cost system which can be used to collect data in situ with high spatial and temporal resolution across remote areas, where no monitoring data are available.

## 4 Experimental Section

### Biological Investigation

*P. appendiculatum* (L.f.) Willd seeds were purchased from Greenmarket di Barbone Valerio, Bergamo Italy. Morphometric analysis of the awns was carried out using a digital caliper (RS PRO 150 mm Digital Caliper 0.0005 in, 0.01 mm, Metric & Imperial, UK) with a resolution of ±0.01 mm and a digital microscope (KH‐8700, Hirox, Japan). The seed mass was measured with an analytical balance (KERN ABS‐N, Germany) with a resolution of ±0.0001 g. Pelargonium awns were separated between active and inactive hygromorphic regions and cut into 5 mm sections. Samples were softened with 4% ethylenediamine for 3–4 days, dehydrated and embedded in paraffin^[^ ^38^ ^]^ and cut into 10 µm sections with a manual microtome (Leica SM2010R, Germany). In the fluorescence analysis, the sections were stained with Fluorescent brightener 28 (Merck, Germany) for cellulose identification, whereas lignin autofluorescence was visible with the blue light excitation (488 nm). Cellulose detection was also achieved with the Alcian blue (Merck, Germany) histochemical stain. Images were captured with Nikon Eclipse Ni‐U optical and fluorescence microscope (Nikon, Japan).

### Pitch and Radius Measurements

A climatic test chamber (CTC256, Memmert GmbH, Germany) was used to investigate the humidity‐responsive deformation of samples in a spatially homogeneous humid air environment. Samples were subjected to humidity ramp at controlled temperature (30 °C) from 30% RH to 90% RH with 10% RH gap, while simultaneously video recording the variation of radius and pitch (Logitech Brio Stream, Logitech, Swiss). To ensure a complete moisture diffusion in hygroscopic active layer, each humidity value was fixed for 5 min (see Supporting Information). The RH inside the chamber was monitored using standard precalibrated humidity meter. To evaluate the variation of geometrical parameters, all the video‐data were post‐processed using ImageJ software.

### Extensional Force and Torque Measurements

Natural and artificial samples were tested in controlled temperature and humidity environment (*T* = 20 °C and RH = 40%), measuring the force through a load cell (Futek LSB200, Futek Advanced Sensor Technology Inc., US) and abruptly increasing the local humidity using a water aerosol. To reduce mechanical vibrations, the load cell was fixed on an optical bench suspended on a passive isolator. A commercial humidity and temperature sensor (Sensirion SHT21) was used to monitor constantly the sample in analysis. A complete description of the experimental setup is provided in Section S3, Supporting Information.

### Diffusion Constant Measurement

Hygroscopic structures had been subjected to a humidity stepwise function from RH = 30% to RH = 90%, (see Section S3, Supporting Information), flowing locally as a water aerosol, and monitoring the variation of geometry using a camera (Logitech Brio Stream, Logitech, Swiss). With the kinematic and morphological analysis, it was possible to determine diffusion time and consequently the diffusion constant.

### Substrate Fabrication

The PCL (Number average molar mass 45 000 g mol^−1^, Sigma Aldrich, US) substrate was produced through fusion deposition modeling (3DBioplotter, EnvisionTEC, Germany), keeping the temperature of hot‐melt extruder at 150 °C, stabilizing the melt polymer for 10 min and using cylindrical nozzle (3 mm length and 0.2 mm internal diameter). The PCL was printed on aluminum foil cleaned in ethanol, then fixed on printing plate at a temperature of 40 °C. To optimize printing condition, the extrusion pressure was set at 6 bar, and the printing speed at 11 mm s^−1^. Nozzle offset was set to 100 µm for beam samples and to 75 µm for artificial seed samples. Printing direction was fixed at 70° with distance between strands of 0.20 mm.

### Surface Activation

To guarantee adhesion with aqueous‐based fibers, an air plasma treatment (Targeo Plasma Cleaner, PIE Scientific, US) was applied on the 3D printed substrate. The plasma time was set to 30 s, gas stability time to 15 s, power setpoint to 150 W, gas setpoint 30 sccm (cm^3^ min^−1^) and base vacuum to 0.50 mbar. To evaluate the effectiveness of the process, a contact angle measurement was done with an optical tensiometer (Theta OneAttension, Biolin Scientific, Sweden) (see Section S3, Supporting Information).

### Nanofiber Deposition

The fiber deposition was carried out by electrospinning technique. The electrospinning apparatus (Linari RT Advanced, Linari Engineering srl) consists in a syringe pump, a coaxial needle (internal diameter 1.2 mm, external diameter 1.8 mm), a rotary drum collector (10 cm diameter), and a high voltage supply. The distance between the end of the coaxial tip and the sample substrate was set to 5 cm, while the collector rotates at 2500 rpm (tangent velocity on collector surface 13.08 m s^−1^). The syringe's vertical speed was set to 1 cm s^−1^ and the voltage applied to the polymer drop was 20 kV (electric field mean modulus 4 kVcm^−1^). The flow rate of CNC core solution and for PEO shell solution were 1 ml/h and 2 ml/h, respectively. All the processes were carried out at RH = 20% and *T* = 20 °C, with a number of round‐trip cycles (RTC) equal to 250 (6 h:30 min). Fiber orientation and diameter analysis were evaluated using ImageJ software^[^ ^39^ ^]^ (see Section S3, Supporting Information).

### Fiber Composition

The solution used for the realization of the core fiber was composed by CNC 5% (Nanografi Nanotechnology AS, Turkey), dispersed in ultrapure water (Millipore Milli‐Q gradient A10, resistivity >18 MΩ·cm^−1^) under continuous stirring (500 rpm) at room temperature for 24 h. The shell component was a 10 wt% aqueous solution of PEO, (viscosity‐average molecular weight 300 000 g mol^−1^, Sigma Aldrich, US) and 10 vol% of ethanol (96% of concentration, Merck, Germany), dispersed under continuous stirring (100 rpm) at *T* = 80 °C for 4 h. The solutions were preserved under chemical hood in continuous stirring, at most for one week.

### Elastic Modulus Measurement

Cap and ridge layer Young's modulus was measured using nanoindentation technique (INano, Nanomechanics Inc., US). Natural samples were investigated applying Oliver–Pharr method,^[^ ^40^ ^]^ using Berkovitch tip (Young's modulus E = 1141 GPa, Poisson's ratio *ν* = 0.07) and applying a target triangular function load of 5 mN, target depth 300 nm, with an indentation strain rate of 0.2%/s. For artificial samples, dynamic nanoindentation^[^ ^41^ ^]^ was done due to the viscoelastic nature of polymers involved. A cylindrical flat tip was used, with a punch diameter of 105 µm. The surface approach distance was fixed at 15 µm, with surface approach velocity of 500 nm s^−1^, applying 100 nm of pre‐test compression (see Supporting Information). During the measurement the temperature was kept at constant humidity RH = 50% and temperature *T* = 25 °C. Moreover, the variation of Young's Modulus of the hygroscopic material in relation to RH using a tensile test was investigated (see Section S3, Supporting Information).

### Morphology of Artificial Structures

Thickness measurements of the 3D printed PCL and the electrospun fiber layer were performed using an optical profilometer (Leica DCM3D, Leica Microsystems, Germany). The fiber layer thickness was evaluated after a localized deposition on flat aluminum substrate, then gently scratched and finally cut using razor blade. SEM images (Zeiss EVO LS10, Germany) were obtained with 10 kV accelerating voltage, after gold sputtering. Optical microscopy was done by Hirox KA 8700 (Japan) digital microscope.

### Statistical Analysis

The normality of data distribution was tested with the Shapiro–Wilk test; normally‐distributed data were analyzed with ANOVA followed by LSD post hoc with Bonferroni correction and expressed as average ± standard error. Non‐normally distributed data were analyzed with the Kruskal–Wallis test followed by pairwise Wilcox post hoc test with Holm correction and expressed as median ±95% confidence interval. Each experiment had been performed in triplicate (*n* = 3), if not differently indicated.

## Conflict of Interest

The authors declare no conflict of interest.

## Author Contributions

L.C. contributed to conceptualization, performed the experiments except for morphometric and histology analyses, validate results through modeling, wrote, edited and reviewed the manuscript; S.M. contributed to climatic chamber tests, performed morphometric and microscopy analyses, provided advisory suggestions, supervised and validate experiments, contributed to the write and review of the manuscript; M.R. provided histological analysis, gave suggestion to biological interpretation, wrote and reviewed the manuscript; A.M. supervised and validate experiments, and reviewed manuscript; N.M.P. validate modeling, revised the manuscript; B.M. directed the research, funding, and revised the manuscript.

## Supporting information

Supporting Information

Supplemental Video 1

Supplemental Video 2

Supplemental Video 3

Supplemental Video 4

Supplemental Video 5

Supplemental Video 6

## Data Availability

The data that support the findings of this study are available from the corresponding author upon reasonable request.
