# Supplementary material for: 4D Printing of Humidity‐Driven Seed Inspired Soft Robots
Source: Adv Sci (Weinh). 2023 Feb 1;10(9):2205146. doi: 10.1002/advs.202205146 (PMC10037692; doi:10.1002/advs.202205146)
Supplement: Supplementary file 1 — Supporting Information [file ADVS-10-2205146-s007.pdf]

Supporting Information

4D printing of humidity-driven seed inspired soft robots

L.Cecchini[1,2], S. Mariani[1], M. Ronzan[1], A. Mondini[1], N.M. Pugno[2,3*] and B. Mazzolai[1*]*
[1] Bioinspired Soft Robotics Laboratory, Istituto Italiano di Tecnologia, Via Morego 30, 16163, Genova
[2] Laboratory for Bioinspired, Bionic, Nano, Meta Materials and Mechanics, Department of Civil, Environmental and Mechanical Engineering, University di Trento, Via Mesiano 77, 38123, Trento
[3] School of Engineering and Materials Science, Queen Mary University of London, Mile End Road, E1 4NS, London, United Kingdom

[*]Emails: stefano.mariani@iit.it nicola.pugno@unitn.it barbara.mazzolai@iit.it

The PDF file includes:
Section S1. *Pelargonium appendiculatum* seed characterization.
Section S2. Biomechanical modeling.
Section S3. Kinematic and Static measurements.
Section S4. Materials characterization.
Section S5. Artificial seed design and processing.

Fig. S1. Morphometric analysis of *Pelargonium appendiculatum* seed.
Fig. S2. Histological analysis of *Pelargonium appendiculatum* awn and lever by bright field and fluorescence microscopy.
Fig. S3. Histological analysis of *Pelargonium appendiculatum* awn and lever by SEM.
Fig. S4. Histological analysis of *Erodium malacoides* awn and lever by bright field and fluorescence microscopy.
Fig. S5. Modeling parameters.
Fig. S6. Temperature and relative humidity variation used for kinematic characterization.
Fig. S7. Experimental setup for extensional force and moment measurements.
Fig. S8. Parameters involved in dynamic measurement.
Fig. S9. Electrospun fibers on Kapton layer.
Fig. S10. Diameter and orientation evaluation of fibers.
Fig. S11. Dynamic nanoindentation of coaxial PEO/CNC electrospun fibers.
Fig. S12. Tensile test of PEO/CNC fiber layer as function of RH.
Fig. S13. Oxygen plasma on PCL substrates.
Fig. S14. Dynamic nanoindentation of FDM PCL layer.
Fig. S15. Fabrication of bilayered hygroscopic actuator.
Fig. S16. Schematic representation of the 4D printing processing.
Fig. S17. Design parameter for artificial seed.
Fig. S18. Fabrication of an artificial seed.
Fig. S19. Pictures of deformation during consecutive humidity cycles for training of the material.
Fig. S20. Thickness evaluation of artificial seed awn.
Fig. S21. Examples of samples produced.
Fig. S22. Experimental setup for extensional force and moment measurements in artificial seed.
Fig. S23. Soil exploration tracking of artificial seed's capsule.
Fig. S24. Experimental evaluation of absolute speed and relative position based on motion tracking.

Table S1. Summary of geometrical, material, mechanical data and results involved in extrapolation of bioinspired elements for design of artificial seed.

Section S1. ***Pelargonium appendiculatum* seed characterization**

Pelargonium appendiculatum seeds were purchased from Greenmarket di Barbone Valerio, Bergamo, Italy. We first proceeded with a morphometric characterization, including analysis of geometrical components and relative weight analysis, as reported in Fig. S1. Weight measurements, either dry or wet, was conducted according to the following protocol: for the drying process, the samples were put in in a climatic chamber at 30 °C with RH = 30% overnight, whereas to obtain the wet weight, the samples were soaked in deionized water for 4h.

For what concerns histological protocol, we have followed Carlquist's ethylenediamine softening and paraffin embedding method (1): it consists of softening small bark tissue samples in 4% ethylene-diamine for three to four days, followed by rinsing in water, dehydration using ethanol and tertiary butanol, and embedding in high-grade paraffin with a 59 °C–61°C melting point. Then, to favor their consolidation, the paraffin blocks are stored in the refrigerator for 3-4 days before cutting. Transverse sections of 10μm thickness were cut, from their respective paraffin blocks, using a microtome (Leica SM2010R). Following the successful sectioning, the samples were placed on glass slides and deparaffinized using a sequence of xylene, ethanol, and pure water (1).

For SEM imaging, the samples are mounted onto aluminum stubs and coated with a 15 nm gold layer using a sputter coater (Quorum Q150R ES, United Kingdom). SEM imaging was carried out with EVO LS 10 (Zeiss, Germany) at a tension of 5KV. Bright field and fluorescence microscopy was conducted with a Nikon Eclipse Ni-U, Japan. Fig. S2 and Fig. S3 report bright field/fluorescence and SEM microscopy analysis, respectively, of the awn and the lever. Fig. S4 report bright field/fluorescence microscopy section analysis of *Erodium malacoides* seed.

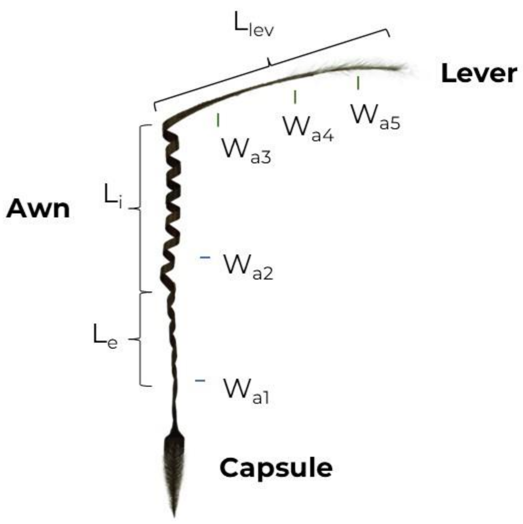

Pelargonium Appendiculatum	
Number Samples Analysed	10
Mass dry (mg)	5.2±0.2
Mass wet (mg)	6.8±0.8
L_i dry (mm)	13.4±0.8
L_i wet (mm)	21.7±1.1
L_e dry (mm)	4.3±0.4
L_e wet (mm)	5.2±0.3
L_{lev} (mm)	17.1±2.3
W_{a1} (um)	258±11
W_{a2} (um)	428±38
W_{a3} (um)	212±21
W_{a4} (um)	138±10
N_c	6-7

Fig. S1. Morphometric analysis of *Pelargonium appendiculatum* seed. The seed is divided into three main components (capsule, awn and lever) and the relative geometrical measurement are reported in the table.

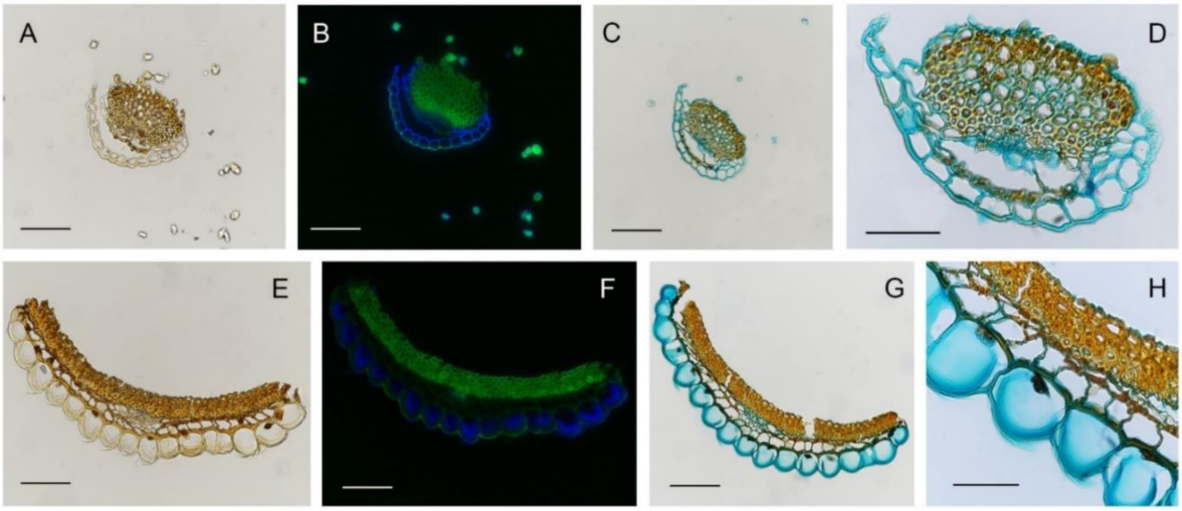

Fig. S2. Histological analysis of *Pelargonium appendiculatum* awn and lever by bright field and fluorescence microscopy. Transversal sections of the lever (a, b, c, d) and awn section (e, f, ,h, g). **a**) Lever bright-field image of a transversal section. **b**) Fluorescent image of (A) with lignin autofluorescence, excitation at 488nm, and staining of cellulose with fluorescent brightener 28. **c-d**) Lever transversal section with cellulose stained with Alcian blue. **e**) White image of a transversal section of the awn in the active region. **f**) Fluorescent image of (E) with lignin autofluorescence, excitation at 488nm, and staining of cellulose with fluorescent brightener 28. **g-h**) Transversal section of the awn with cellulose stained with Alcian blue. Scalebar is 500µm for (a, b, c, e, f, g). Scalebar is 50µm for (d, h).

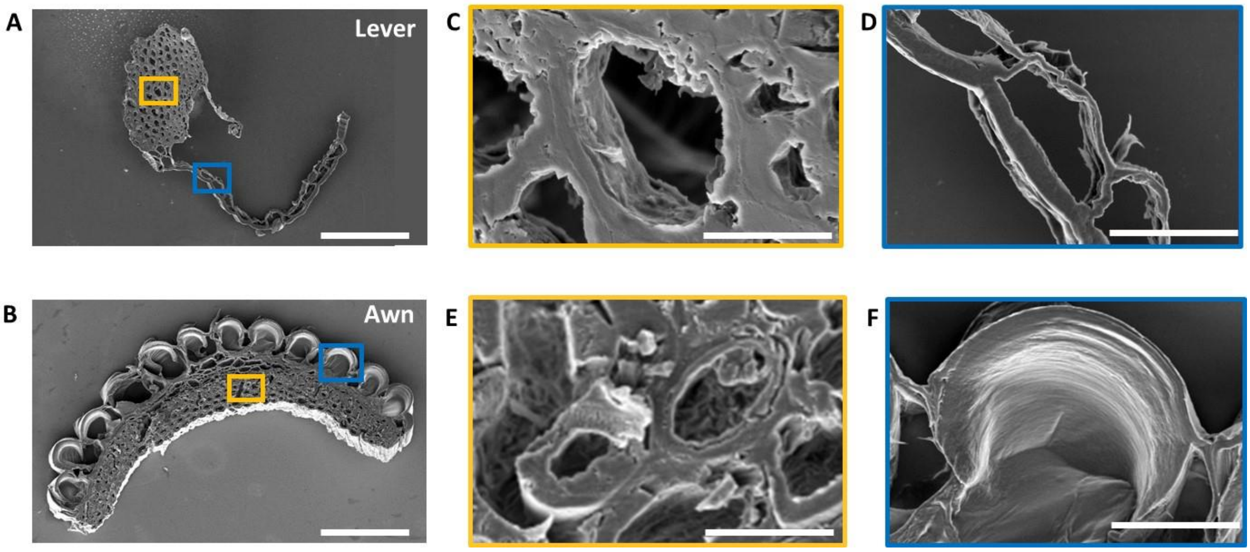

Fig. S3. Histological analysis of *Pelargonium appendiculatum* awn and lever by SEM. SEM sections of the *Pelargonium appendiculatum* awn lever a) and in the awn section b) c) and d) are zooms taken from image a) while e) and f) are zoom taken from image b). Scalebars is 100 µm for a) and b), 10 µm for c) and e) and 20 µm for d) and f).

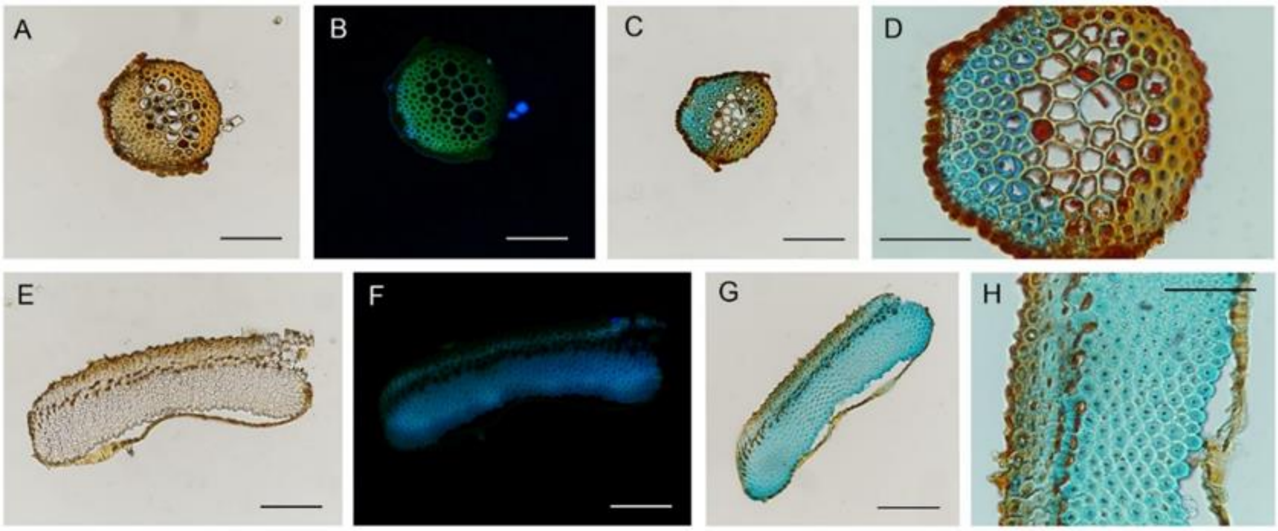

Fig. S4. Histological analysis of *Erodium malacoides* awn and lever by bright field and fluorescence microscopy. Transversal sections of the lever (a, b, c, d) and awn (e, f, g, h). a) White image of a transversal section of the lever. b) Fluorescent image of A with lignin autofluorescence, excitation at 355 and 488nm. c) Transversal section of the lever stained with cellulose with Alcian blue. d) Magnification of image c. e) White image of a transversal section of the awn. f) Fluorescent image of E with lignin autofluorescence and staining of cellulose with fluorescent brightener 28, excitation at 355 and 488nm. g) transversal section of the awn in the active region with cellulose stained with Alcian blue. h) Magnification of image G. Bars= 500µm (a, b, c, e, f, g), 50µm (d, h).

Section S2. Biomechanical modeling
In 2020, Ha et al. (2) explained the overall awn deformation of *P. appendiculatum* considering theory of laminated composite plates, where only the thickness participates in deformation among geometrical parameters.
The deformation tendencies of the plates depends on the stress generated by hygroscopic expansion and a simple constitutive relation can be expressed as:

$$\boldsymbol{\sigma} = \sum_i \boldsymbol{D_i} \left(\boldsymbol{\epsilon} - \boldsymbol{\epsilon_{h_i}}\right)$$

where where i represents the i-th layer, $\mathbf{D_i}$ the material stiffness and $\boldsymbol{\epsilon}_\mathbf{h}$ the hygroscopic strain associated to the i-th layer, modeling the hygroscopic driving effect as detailed below, $\boldsymbol{\sigma}$ is the stress and $\boldsymbol{\epsilon}$ the strain.

Considering the z as the direction along the thickness of the seed, it is possible to describe the total strain at distance z from the bottom plane $(z_0 = 0)$ as $\boldsymbol{\epsilon}(z) = \boldsymbol{\epsilon_0} + \boldsymbol{\kappa} z$ under Kirchoff assumption, where $\boldsymbol{\epsilon_0}$ is the bottom plane strain and $\boldsymbol{\kappa}$ the curvature. The constitutive relation leads to the matrix equation of forces and moments:

$$\mathbf{F} = \mathbf{A}\epsilon_0 + \mathbf{B}\,\kappa$$

$$\mathbf{M} = \mathbf{B}\epsilon_0 + \mathbf{C}\,\kappa$$

where ***F*** is the force matrix, **M** the moment matrix, **A** the extensional stiffness, **B** the coupling stiffness and **C** the bending stiffness defined as:

$$\mathbf{A} = \sum_{k=1}^{n} \mathbf{D}_k (z_{k+1} - z_k)$$
$$\mathbf{B} = \frac{1}{2}\sum_{k=1}^{n} \mathbf{D}_k \left(z_{k+1}^2 - z_k^2\right)$$
$$\mathbf{C} = \frac{1}{3}\sum_{k=1}^{n} \mathbf{D}_k \left(z_{k+1}^3 - z_k^3\right)$$

where k is the k-th layer definition and **D** is the stiffness matrix defined as:

$$\mathbf{D} = \mathbf{R}\,\mathbf{H}^{-1}\boldsymbol{R}^{-1} \begin{pmatrix} \frac{E_{11}}{1-\nu_{12}\nu_{21}} & \frac{E_{11}\,\nu_{21}}{1-\nu_{12}\nu_{21}} & 0 \\ \frac{E_{22}\,\nu_{12}}{1-\nu_{12}\nu_{21}} & \frac{E_{22}}{1-\nu_{12}\nu_{21}} & 0 \\ 0 & 0 & G_{12} \end{pmatrix}$$

where 1 and 2 represents respectively parallel and normal direction of the fibres, E is the Young's modulus, ν is the Poisson's ratio, G is the shear modulus and **H** and **R** are respectively transformation matrix and Reuter's matrix, where the cellulose microfibril angle is in relation to the cellulose helix axis θ (MFAH):

$$\mathbf{H} = \begin{pmatrix} \cos^2(\theta) & \sin^2(\theta) & 2\sin(\theta)\cos(\theta) \\ \sin^2(\theta) & \cos^2(\theta) & -2\sin(\theta)\cos(\theta) \\ -\sin(\theta)\cos(\theta) & \sin(\theta)\cos(\theta) & \cos^2(\theta) - \sin^2(\theta) \end{pmatrix}$$

$$\mathbf{R} = \begin{pmatrix} 1 & 0 & 0 \\ 0 & 1 & 0 \\ 0 & 0 & 2 \end{pmatrix}$$

Since **F** and **M** are induced by hygroscopic swelling, the total strain can be modelled according to the definition of hygroscopic expansion coefficient (CHE), that represents the amount of strain generated per percentage unit of relative humidity.
So, the hygroscopic stress can be expressed as $\boldsymbol{\epsilon_h} = \boldsymbol{\alpha}\,\Delta\Phi = \boldsymbol{\alpha}\,(\Phi - \Phi_0)$, where $\boldsymbol{\alpha}$ is the linear coefficient of hygroscopic expansion, Φ is the relative humidity and Φ_0 is the value of relative humidity at which the mechanical system shows a curvature $\boldsymbol{\kappa} = \mathbf{0}$. To calibrate the considered model, Φ_0 must be evaluated experimentally (as a working parameter accounting for, e.g., residual stresses and structural imperfections resulting from fabrication). The vectorial CHE is described as:

$$\boldsymbol{\alpha} = \begin{pmatrix} \alpha_{xx} \\ \alpha_{yy} \\ \alpha_{xy} \end{pmatrix} = \boldsymbol{R}\boldsymbol{H}^{-1}\boldsymbol{R}^{-1} \begin{pmatrix} \alpha_{11} \\ \alpha_{22} \\ \alpha_{12} \end{pmatrix}$$

So, it is possible to represent **F** and **M** considering the definition of hygroscopic strain and supposing that it is the only (internal) force acting on the structure:

$$\mathbf{F} = \int_0^{h} \mathbf{D}\,\boldsymbol{\alpha}\,\Delta\phi\,\mathrm{dz}$$
$$\mathbf{M} = \int_0^{h} \mathbf{D}\,\boldsymbol{\alpha}\,\Delta\phi\,\mathrm{z}\,dz$$

where h is the whole thickness of the n-th layer and $\Delta\phi$ is the relative humidity (RH) variation.
Since material properties are independent of z-axis (neglecting the discontinuity points or considering piecewise-continuous functions such that $d\boldsymbol{D}/dz = 0$ and $\mathrm{d}\boldsymbol{\alpha}/\mathrm{dz} = 0$), we can express **F** and **M** as:

$$\mathbf{F} = \sum_{k=1}^{n} \mathbf{D}_k \boldsymbol{\alpha_k}\,\Delta\phi(z_{k+1} - z_k)$$
$$\mathbf{M} = \frac{1}{2}\sum_{k=1}^{n} \mathbf{D}_k \boldsymbol{\alpha_k}\,\Delta\phi(z_{k+1}^2 - z_k^2)$$

Now, the strain and the curvature vector are obtained considering the definition of **F** and **M**:

$$\begin{pmatrix} \boldsymbol{\epsilon_0} \\ \boldsymbol{\kappa} \end{pmatrix} = \begin{pmatrix} \mathbf{A} & \mathbf{B} \\ \mathbf{B} & \mathbf{C} \end{pmatrix}^{-1} \begin{pmatrix} \mathbf{F} \\ \mathbf{M} \end{pmatrix}$$

The curvature vector $\boldsymbol{\kappa} = \left(\kappa_{xx}\ \kappa_{yy}\ \kappa_{xy}\right)^{\boldsymbol{T}}$ can be transformed in the matrix form:

$$\boldsymbol{b} = \begin{pmatrix} \kappa_{xx} & \kappa_{xy} \\ \kappa_{xy} & \kappa_{yy} \end{pmatrix}$$

From the Kirchoff's theory of thin plates, the maximum eigenvalue of the curvature tensor **b** represents the principal curvature κ_0, which determines the helix of shape. The radius R and the pitch P of the helix are finally calculated as:

$$R = \frac{1}{\kappa_0}$$

$$P = \frac{2\pi\, tan(\Psi)}{\kappa_0}$$

where the tilt angle Ψ is the angle between the cellulose helix axis and the cell's long axis.
Since the structure is subjected to bending and twisting moment, the combined effect give rise to the typical coiled structure described above.

It is now possible to evaluate the force and moment generated by the structure considering the theory of mechanical springs subjected to large deformations (3). Considering the hygroscopic actuator as a homogeneous helical cylindrical spring with rectangular cross-section, it is possible to calculate the extensional force F and the moment M_0 in closed form. It is shown from the theory of elasticity that the change in a bar or wire curvature as the spring deflects from an initial pitch angle β_0 to a different pitch angle β is:

$$\Delta\kappa = \frac{\cos^2\beta}{R} - \frac{\cos^2\beta_0}{R_0} \qquad (I)$$

$$\Delta\theta = \frac{\sin\beta\cos\beta}{R} - \frac{\sin\beta_0\cos\beta_0}{R_0} \qquad (II)$$

where $\Delta\kappa$ is the change in curvature of the wire, $\Delta\theta$ is the angle twist per unit length, β is the pitch angle defined from geometrical consideration as:

$$\beta = \arctan\left(\frac{P}{4R}\right) = \arctan\left(\frac{\pi\tan\Psi}{2}\right)$$

The moment M_0 and the load F are acting on the spring simultaneously, so the bending and twisting moment m_b and m_t acting on the wire will be:

$$m_b = M_0\ cos\beta - FR\ sin\beta \qquad (III)$$

$$m_t = M_0\ sin\beta + PR\ cos\beta \qquad (IV)$$

The change in curvature of the wire due to the bending moment is equal to the moment divided by the flexural rigidity EI, where E is the Young's modulus and I is the moment of inertia of rectangular cross section:

$$\Delta\kappa = \frac{m_b}{EI} = \frac{12\, m_b}{E\, b\, h^3} \qquad (V)$$

where b is the width and h the thickness. Moreover, the change in twist angle per unit length due to twisiting moment is equal to moment divided torsional rigidity of rectangular cross section Gk_sbh^3, where G is the shear modulus, k_s the shape factor as a function of b/h:

$$\Delta\theta = \frac{m_t}{G\, k_s b\, h^3} \qquad (VI)$$

Combining Eq. (*I, II, III, IV, V, VI*), we finally obtain:

$$F(\phi) = \frac{Gk_sbh^3 \cos\beta}{R(\phi)}\left(\frac{\sin\beta\cos\beta}{R(\phi)} - \frac{\sin\beta_0\cos\beta_0}{R_0}\right) - \frac{E\, bh^3 \sin\beta}{12\, R(\phi)}\left(\frac{\cos^2\beta}{R(\phi)} - \frac{\cos^2\beta_0}{R_0}\right)$$

$$M_0(\phi) = Gk_sbh^3 \sin\ \beta\left(\frac{\sin\beta\cos\beta}{R(\phi)} - \frac{\sin\beta_0\cos\beta_0}{R_0}\right) + \frac{E\, bh^3 \cos\beta}{12}\left(\frac{\cos^2\beta}{R(\phi)} - \frac{\cos^2\beta_0}{R_0}\right)$$

Note that in this case we assume that the material can be simplified as a homogeneous structure by means of rule of mixtures, where the material properties of the composite are evaluated considering the volume fraction of the fiber layer with respect to the substrate layer.

Knowing the kinematic variation of the geometrical features involved in hygroscopic coiling process (radius and pitch as function of RH) it is finally possible to estimate in a closed-form solution the dynamic parameters that we used to design the artificial seed.
Moreover, considering that the extension phenomenon in helical springs is limited by maximum torsional stress, we can calculate the maximum force that can be generated by structure without yielding.

As reported in literature (4), the maximum allowable torsional stress τ_{Max} for nonferrous alloy in helical compression spring is 35% of tensile strength σ_{Max}. Shape factor of helical compression spring is directly related to b/h aspect ratio. In our case b/h ~ 5 and so, k_s = 0.291 (3), obtaining:

$$P \le P_{Max} = \frac{k_s b^2 h\, \tau_{Max}}{R} = 0.35 \times 0.291 \times \frac{b^2 h\, \sigma_{Max}}{R}$$

Water diffusion in hygroscopic layer is mediated by Fick's laws of diffusion. To predict experimental results, we consider one dimensional problem of diffusion along the thickness z. The choice of this variable is related to the one-dimensional assumption in kinematic model. We now consider the problem of constant concentration source (constant flux in water aerosol for the experimental case) $\Delta\phi$ located at the top of cap layer $z_0 = 0$, with initial concentration at time t_0 equal to ϕ_0 . Considering the boundary conditions (experimental conditions), the one-dimensional diffusion equation admit solution and it is equal to:

$$\phi(z,t) = \Delta\phi\, erfc\left(\frac{z}{2\sqrt{Dt}}\right) + \phi_0$$

where D is the diffusion coefficient and t the diffusion time.

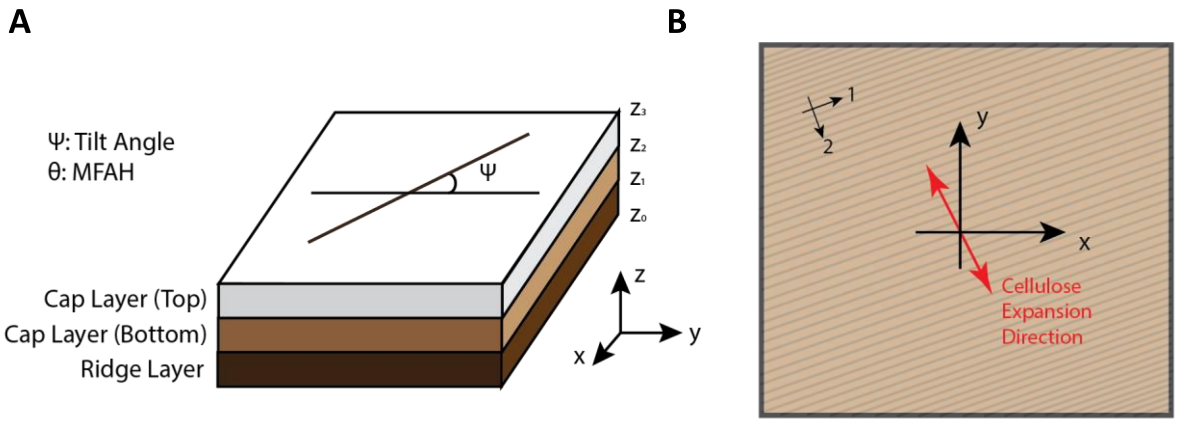

Fig. S5. Modeling parameters. (**a**) Schematic representation of equivalent trilayer decomposition of *Pelargonium appendiculatum*'s awn. (**b**) Relative expansion direction of cellulose microfibrils.

Section S3. Kinematic and Static measurements

We refer as kinematic test the evaluation of macroscopic geometrical features that allows to describe coiling deformation, as a superposition of bending and torsion. We calculate relative humidity variation of pitch and radius in climatic chamber (CTC256, Memmert GmbH), fixing temperature T = 30°C. The choice of temperature value was chosen to guarantee the maximum humidity dynamics of the machine, that ranges from 30% to 90%. Experimental values of RH and T are provided in Fig.S6.

After comparing kinematics with modelling results, we were able to determine the diffusivity of active fiber layer. The procedure consists in: increasing abruptly humidity level using a water aerosol, as reported in Fig.S8B; collect visual data with camera; analyse data with software ImageJ in order to determine diffusion time comparing video results with kinematic analysis provided before.

Finally, we refer as dynamic analysis the evaluation of extensional force and moment in sample. Fig.S7 shows the experimental setup used in these measurements. The experimental setup is composed by optical bench suspended on a foam passive isolator, on which it is fixed 10g sensitive load cell (Futek LSB200). To reduce buckling and perturbation on the measurement, the load cell was covered with sandpaper (coarse grit 150). Load cell calibration was provided in Fig.S8A, using as reference the sample measured with microbalance (Sartorius, Practum 224-1x). Both in extensional force and torque measurements samples were first grounded and then we apply water aerosol. For extensional force measurements, we razor blade cut the awn region of the seed and then we fixed them on measurement plates using hot glue, as reported in Fig. S7B,D. For torque measurement we evaluated the force at the end of the lever, and considering the angle of incidence equal to 90°, we extrapolate the moment as $M_0 \cong F_{Measured}\, r_{Lever}$. All the measurement were monitored by a humidity and temperature sensor. Force measurements are filtered using moving average filter, with a sample window equal to 100.

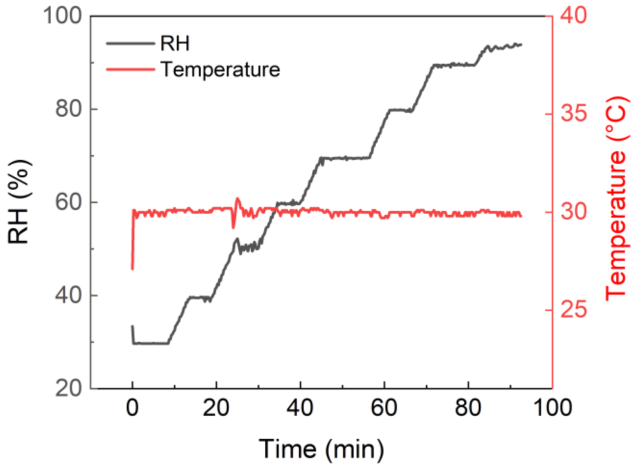

Fig. S6. Temperature and relative humidity variation used for kinematic characterization.

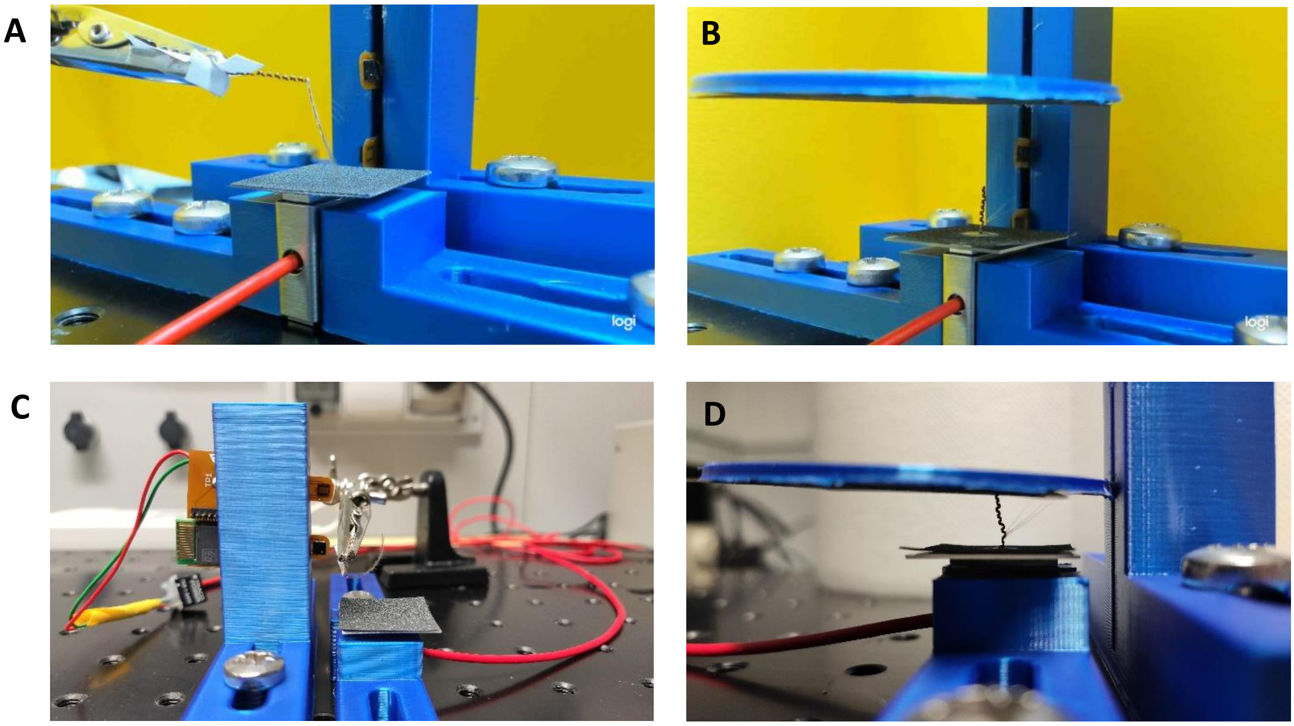

Fig. S7. Experimental setup for extensional force and moment measurements. a) Torque measurement b) Extensional force sample disposition. c) General setup for torque measurement. d) General setup for measurement of extensional force

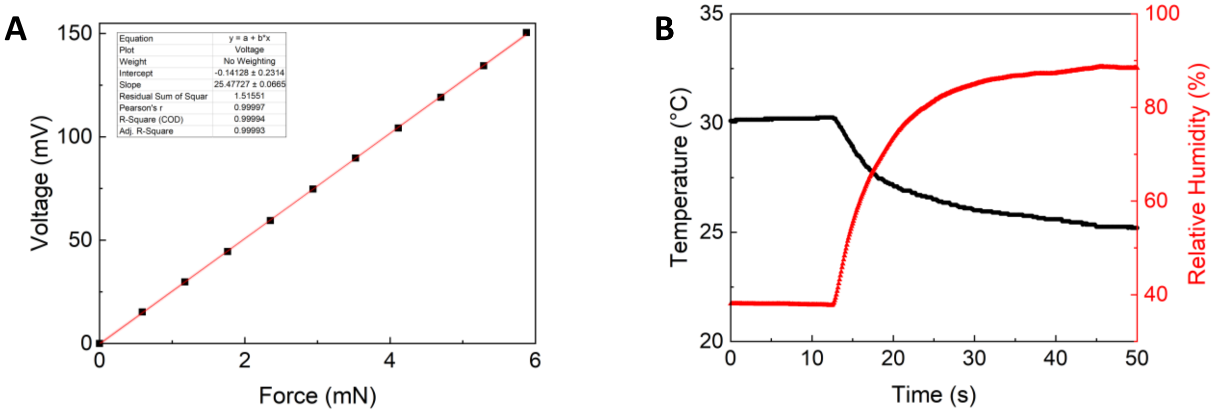

Fig. S8. Parameters involved in dynamic measurement. a) Calibration curve of Load Cell. b) Dynamic variation of temperature and humidity due to water aerosol used for extensional force and moment characterization.

Section S4. Materials characterization

Cellulose nanocrystals were bough from Nanografi (https://nanografi.com/popular-products/cellulose-nanocrystal-nanocrystalline-cellulose-cnc/). They are produced by sulfuric acid hydrolysis without any binder. The dry powder (4% wt. moisture) is composed by 10-20 nm wide, 300-900 nm length 1D nanocrystals, with cellulose crystallinity of 92%. We dispersed the commercial powder in a DIW solution 5% wt. For fiber analysis we used optical microscope images elaborated with ImageJ software. To better understand the diameter distribution and orientation of fibers, we electrospun 1 Round Trip Cycle (RTC), 5RTC and 10RTC on Kapton tape. Then, following standard procedure reported in the literature (5), we put samples in the oven at 40 °C overnight to allow complete desorption of water component. Finally, we evaluated fiber orientation on 10RTC sample, transforming the image in frequency domain by means of 2D FFT and then analysed the distribution with an oval profile (ImageJ https://imagej.nih.gov/ij/plugins/oval-profile.html).

Tensile stress for the evaluation of Young's modulus of PEO/CNC fibers as a function of relative humidity was provided in climatic chamber (T = 30 °C). We realized an *ad hoc* experimental setup, which consist in microtranslational stage combined with microcontroller, to extend samples and evaluate relative precision with micrometric resolution. Dumbell specimen of PEO/CNC fiber layer was produced using electrospinning and consequently laser cut. Relative dimensions of specimen were length = 30mm width = 5mm and thickness = 70µm. Force measurements were provided by Futek LSB201 10lb load cell, in tensile mode. Load cell was previously calibrated. According with tensile stress provided by ZwickRoell Z100, we increased strain by 0.05 mm and we evaluated the relative force, considering as idle force F = 0.100 N. The test was repeated for different values of humidity. We tested the effect of oxygen plasma on FDM printed PCL samples, considering 3 different levels of plasma power. Increasing power up to 150W, the samples started to melt due to local increase of temperature and to low melting point of PCL (60°C).

We then provided a contact angle measurement to understand the effectiveness of the treatment. Experimental conditions are provided in data section.

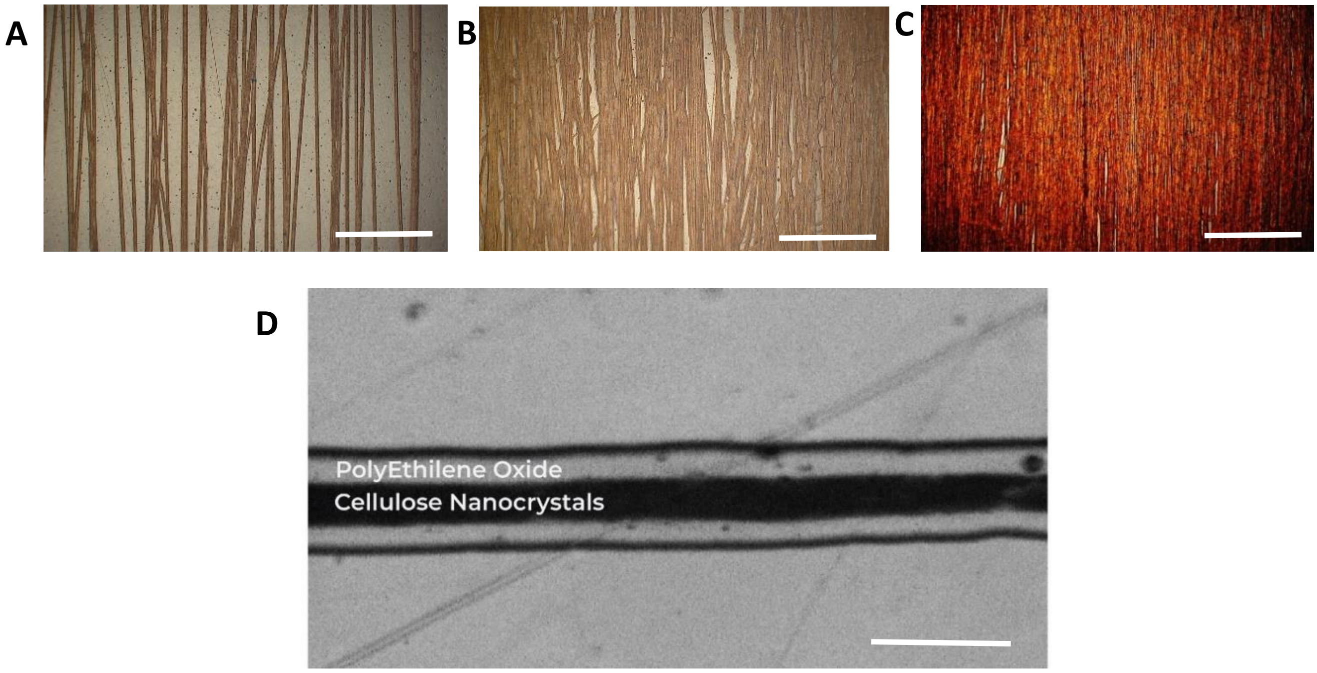

Fig. S9. Electrospun fibers on Kapton layer a) 1RTC deposited fibers. b) 5RTC deposited fibers. c) 10RTC deposited fibers. Scalebars are 500 µm. d) Single coaxial fiber. Scalebar is 20 µm.

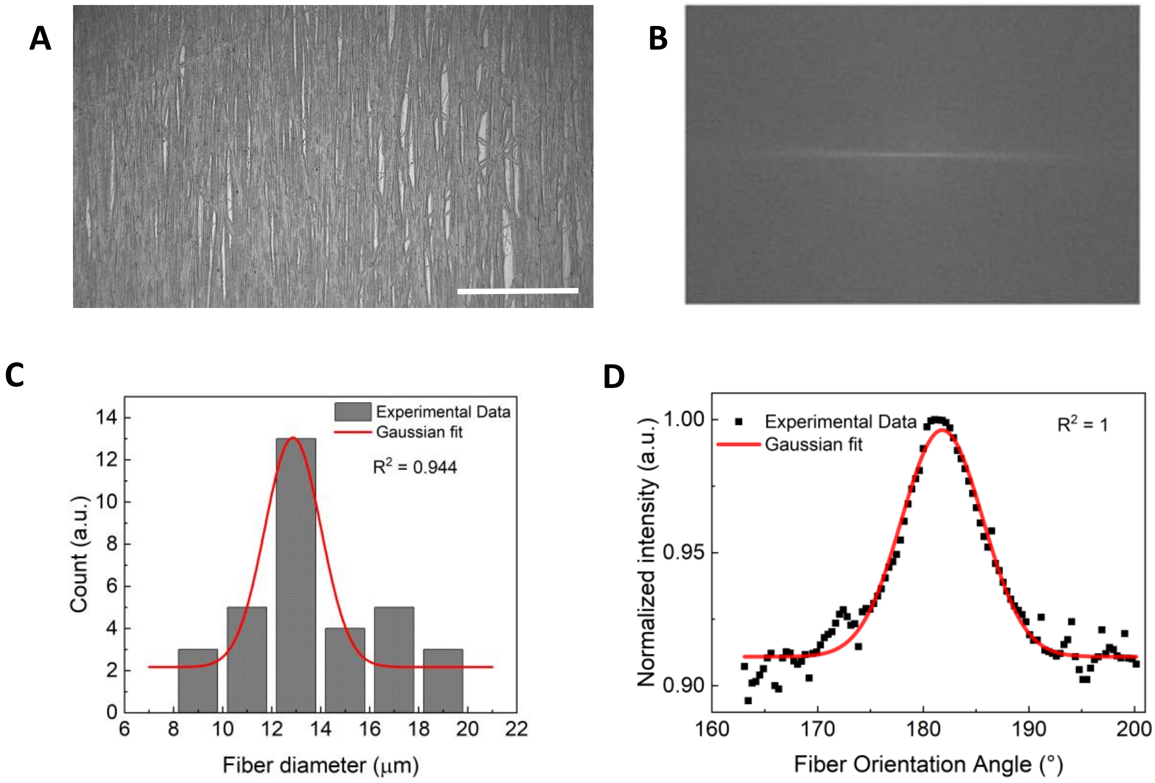

Fig. S10. Diameter and orientation evaluation of fibers a) Grayscale conversion of 10RTC sample. Scalebar is 500 µm. b) 2D FFT transform of Fig.S10A c) Manual evaluation of fiber diameter, with gaussian fit. d) Oval profile evaluation of fiber orientation.

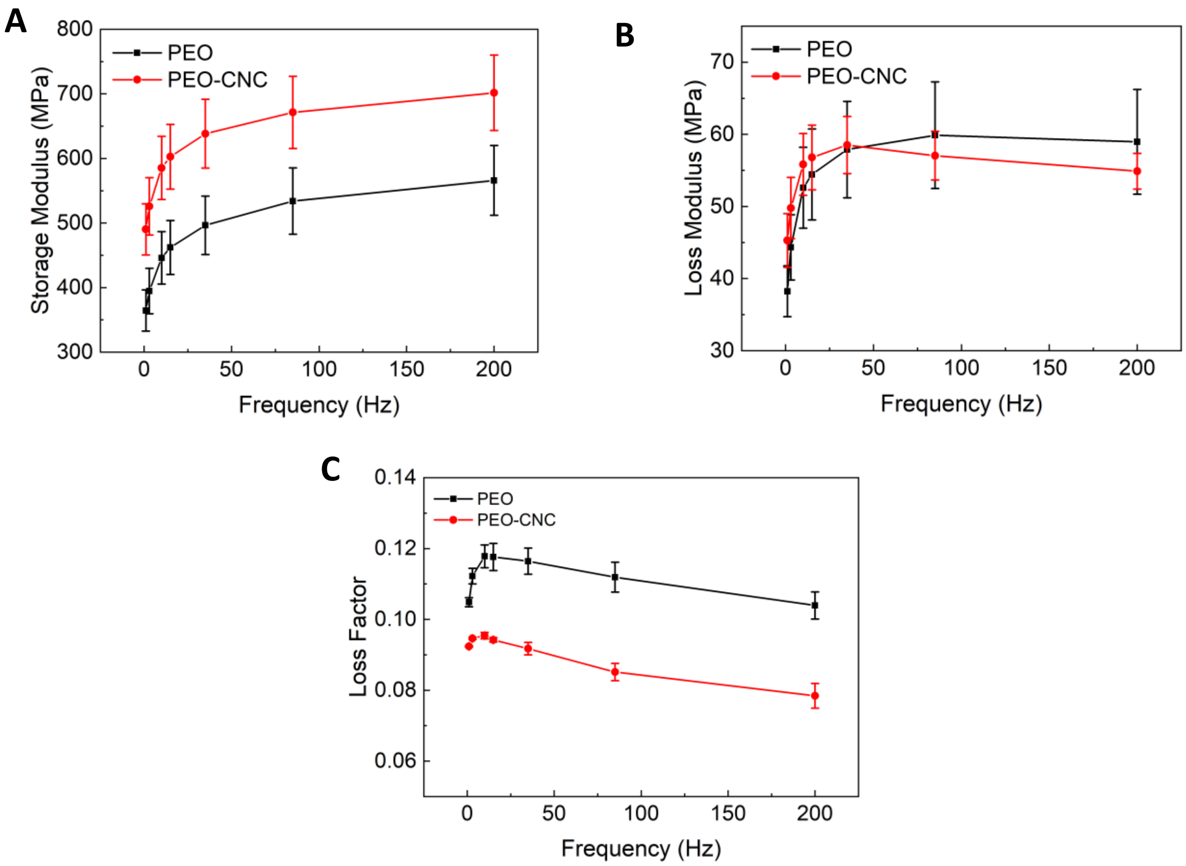

Fig. S11. Dynamic nanoindentation coaxial PEO/CNC electrospun fibers. a) Storage modulus PEO/CNC fiber layer. b) Loss modulus PEO/CNC fiber layer. c) Loss factor PEO/CNC fiber layer. d) Loss modulus PCL layer. e) Storage modulus PCL layer. f) Loss factor PCL layer.

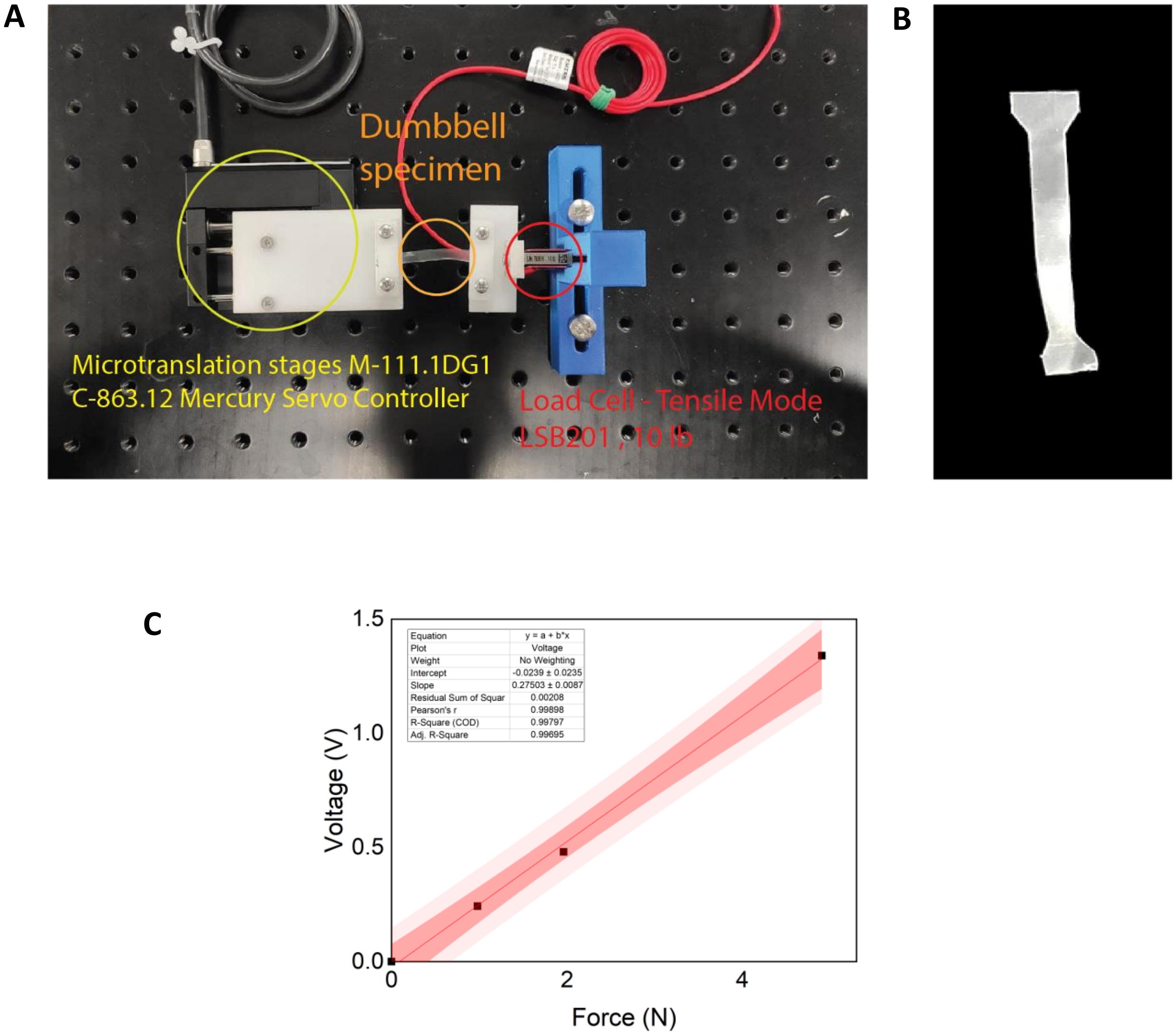

Fig. S12. Tensile test of PEO/CNC fiber layer as function of RH. a) Schematic of experimental setup. b) PEO/CNC dumbbell specimen. c) Load cell calibration.

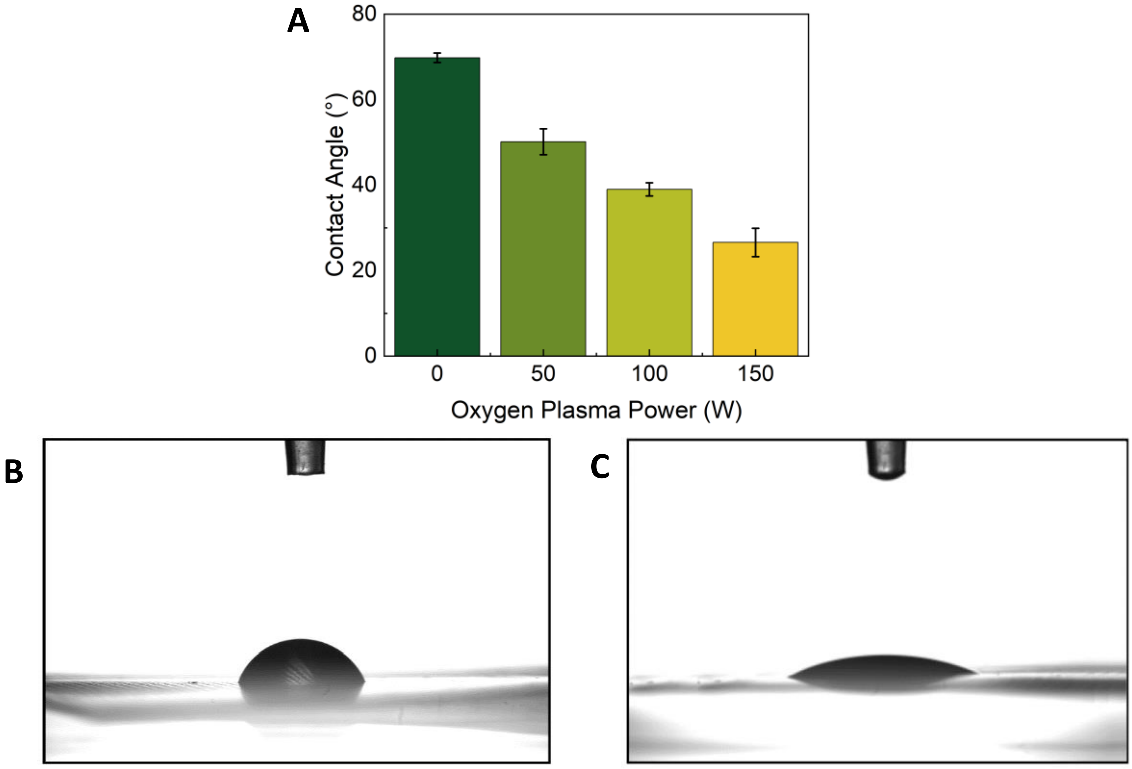

Fig. S13. Oxygen plasma on PCL substrates. a) Contact angle measurements on PCL samples. b) Water droplet on untreated PCL sample. c) Water droplet on 150W oxygen plasma treated PCL sample.

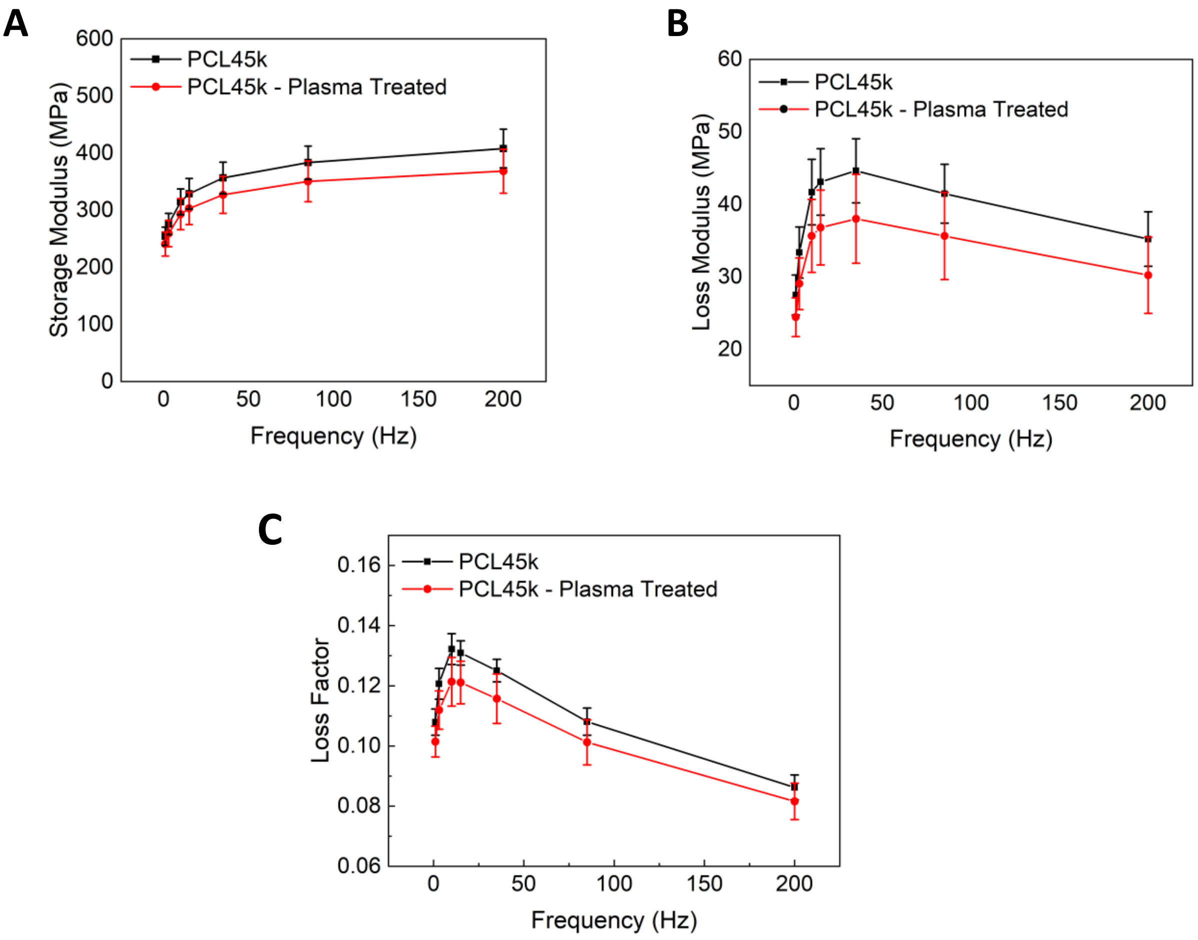

Fig. S14. Dynamic nanoindentation of FDM PCL layer. a) Storage modulus PCL layer. b) Loss modulus PCL layer. c) Loss factor PCL layer.

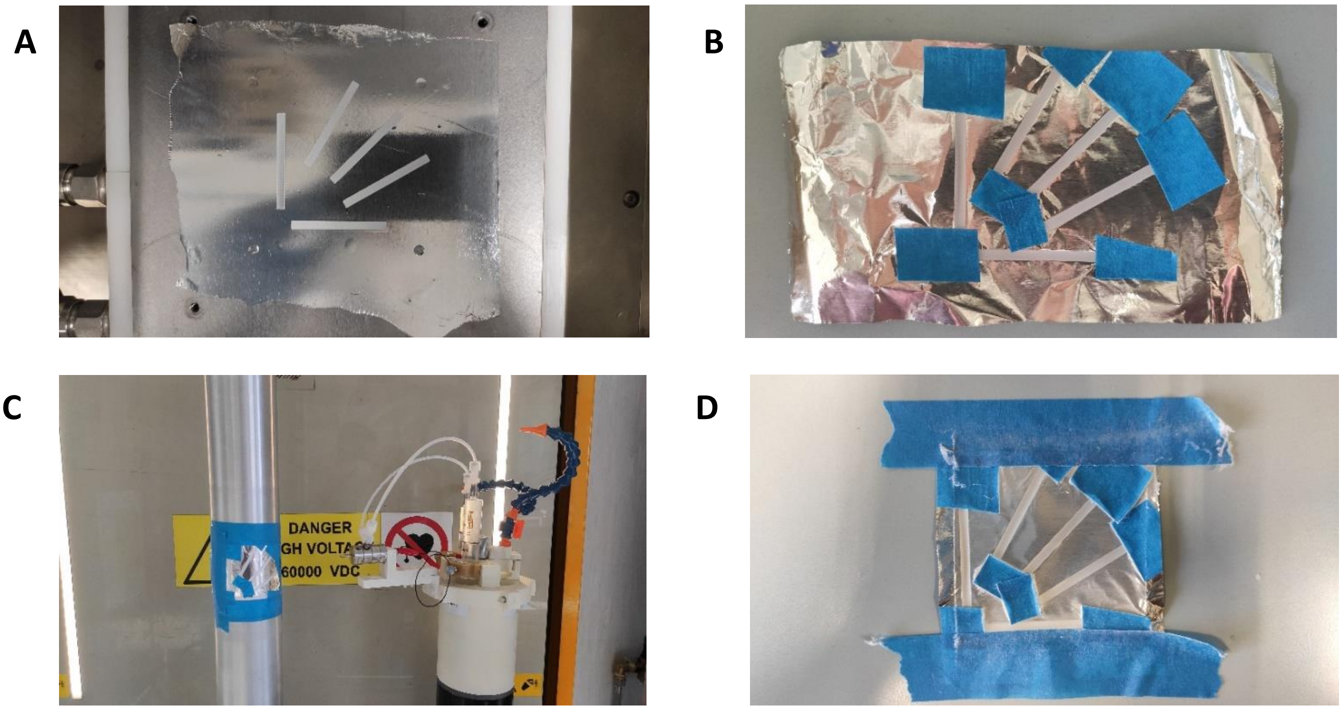

Fig. S15. Fabrication of bilayered hygroscopic actuator a) FDM print of PCL beams. b) Fixing samples on aluminium substrate to prevent detachment during electrospinning process. c) Adaptation of substrates on cylindrical mandrel for electrospinning. d) Samples processed after electrospinning. Samples are oriented in different dispositions to modify selectively the tilt angle.

Section S5. Artificial seed design and processing

The seed design was provided to mimicking geometrical features and mechanical performances of the natural seed. Note that during the design process we took into consideration the relative variation of design dimensions due to FDM processing. We set as printing offset 75µm to reach 100µm real thickness and width 1.3 mm to obtain 1.5 mm during the printing process. This was observed because we used a 200µm nozzle for the realization of passive substrate and so, we worked under the machine resolution. The repeatability of printing process was investigated with statistical analysis. As it possible to observe in Fig.S17, the awn component of artificial seed was set to 15° with respect to capsule direction because of longitudinal expansion of PEO/CNC fibers. Note also that we select as printing direction 70°, mimicking the MFAH role in natural seed. Seed .stl was provided in data section. Motion tracking of seed's capsule was evaluated with Kinovea software. Capsule was targeted with red colour to better improve contrast and consequently to increase accuracy in motion prediction. Since the dynamic evolution of the system is mediated by humidity cycles, we reported data as a function of RH. Speed, y-axis position, x-axis position data are filtered with a moving average filter, sample window equal to 10.

To evaluate the energy density in natural and artificial samples, we use the definition of work and the integration by substitution considering as changing variable relative humidity $\phi(t)$:

$$W = \int_{\theta_i}^{\theta_f} M\, d\theta + \int_{\delta_i}^{\delta_f} F\, dl \approx \sum_{i=\phi_0}^{\phi_{f-1}} M_i\, (\theta_{i+1} - \theta_i) + F_i\, (N_{Coil_{i+1}} P_{i+1} - N_{Coil_i} P_i)$$

where M is the moment, F the extensional force, θ the uncoiling angle, N_{Coil} the number of windings and P the pitch.

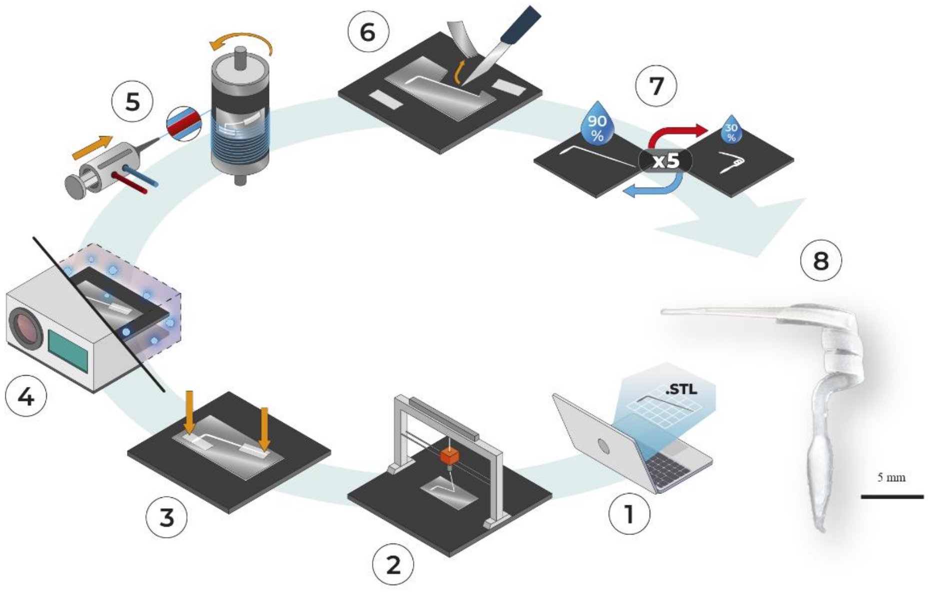

Fig. S16. Schematic representation of the 4D printing processing. (1) Design of the seed with CAD software for .stl production. (2) FDM printing of PCL on aluminum foil paper. (3) Adapting tape on artificial capsule and lever section. (4) Surface activation through oxygen plasma of artificial awn. (5) Hygroscopic functionalization of the artificial awn through coaxial electrospinning of PEO/CNC. (6) Blade cut of the structure from aluminum-foil paper. (7) 4D deformation in a climatic chamber through 5-times humidity cycles. (8) Example of 4D printed artificial seed. Scalebar is 5 mm.

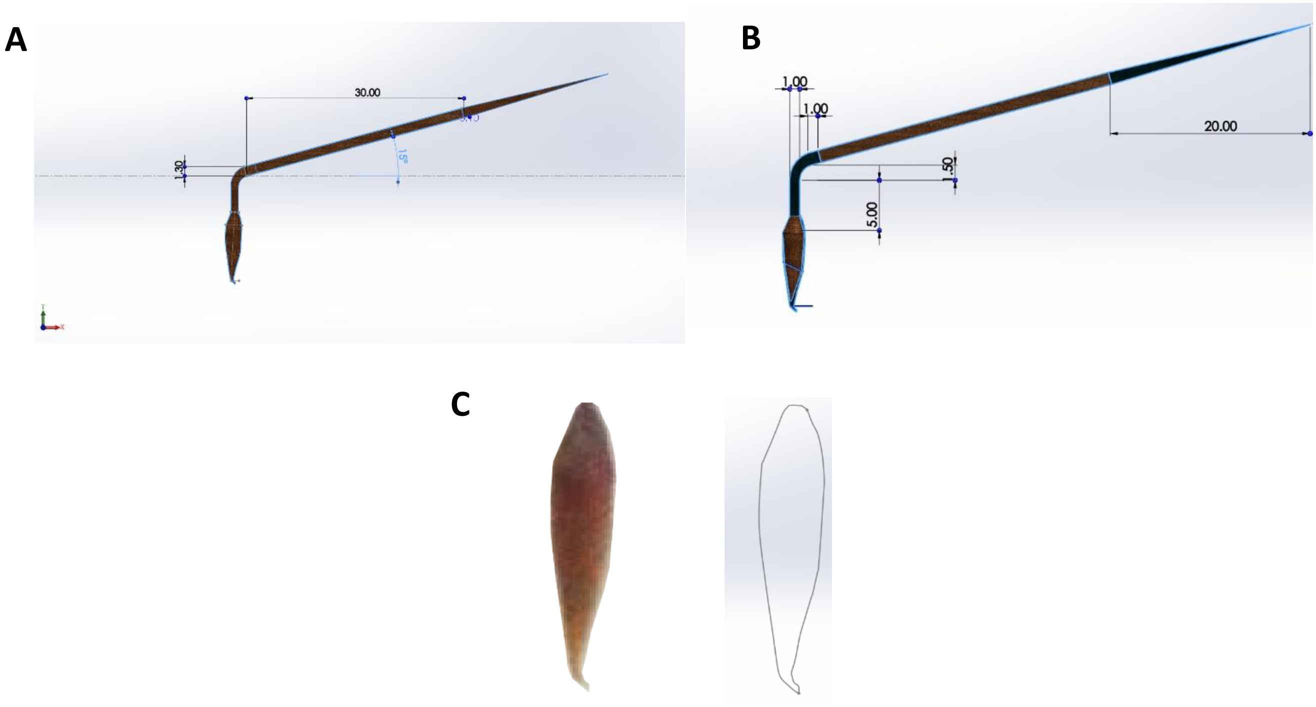

Fig. S17. Design parameter for artificial seed. a) Design of the awn. b) Design of linking elements between capsule and lever, considering material deformation and expansion during FDM process. c) Capsule design according to natural seed capsule, selecting contour plot and change relative dimension to guarantee same aspect ratio with respect to *P. appendiculatum*.

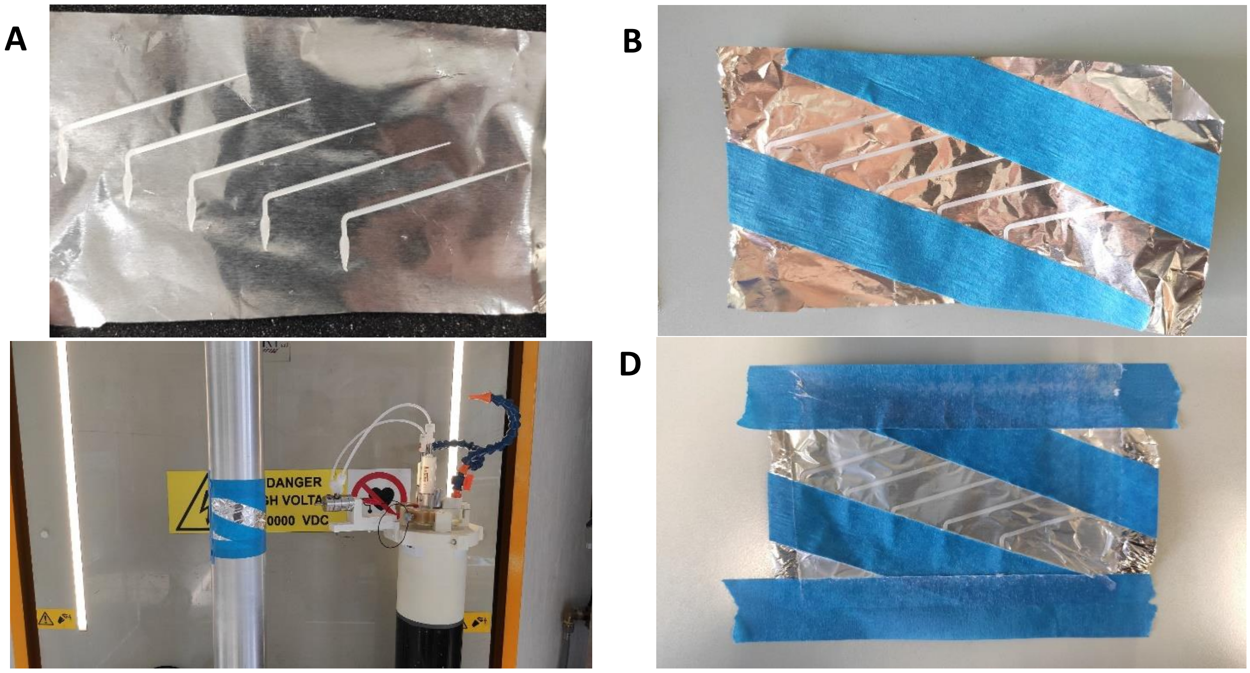

Fig. S18. Fabrication of artificial seed a) FDM print of PCL inactive layer. b) Fixing samples on aluminium substrate to prevent detachment during electrospinning process and to isolate passive regions (capsule and lever). c) Adaptation of substrates on cylindrical mandrel for electrospinning. d) Samples processed after electrospinning.

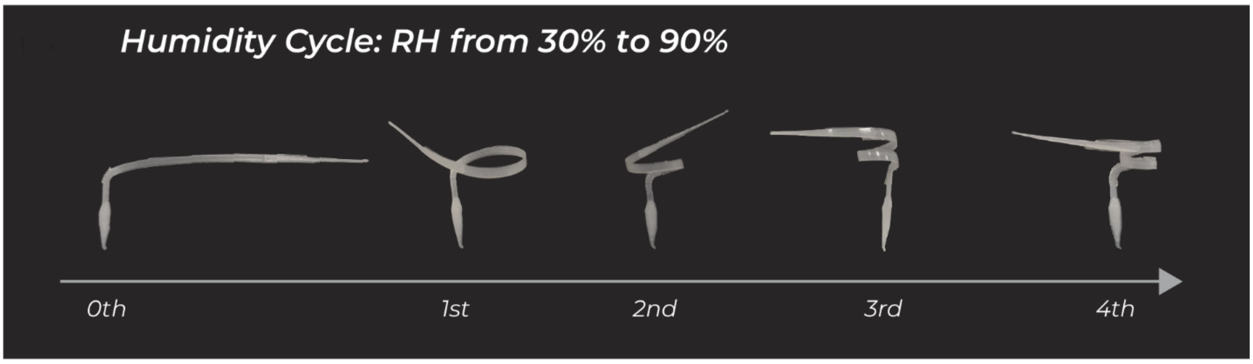

Fig. S19. Pictures of deformation during consecutive humidity cycles for training of the material.

A

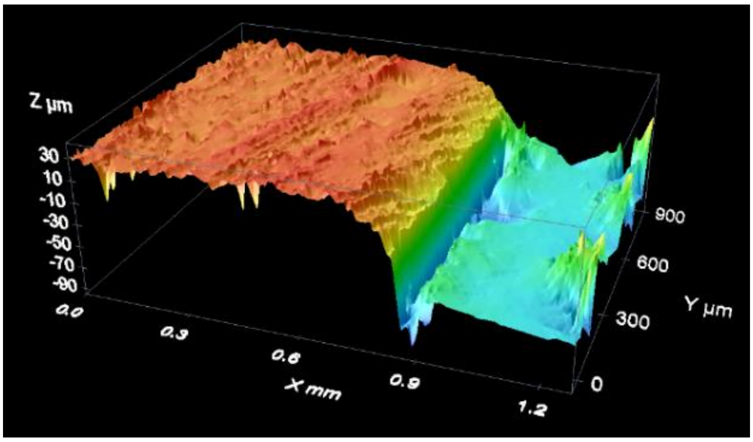

B

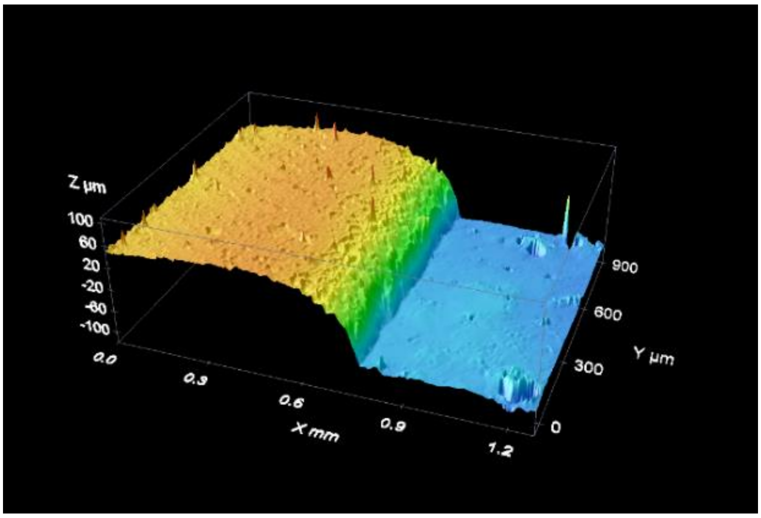

Fig. S20. Thickness evaluation of artificial seed awn. a) Topography of PEO/CNC fiber layer, obtained with optical profilometry. b) Topography of PCL awn, obtained with optical profilometry.

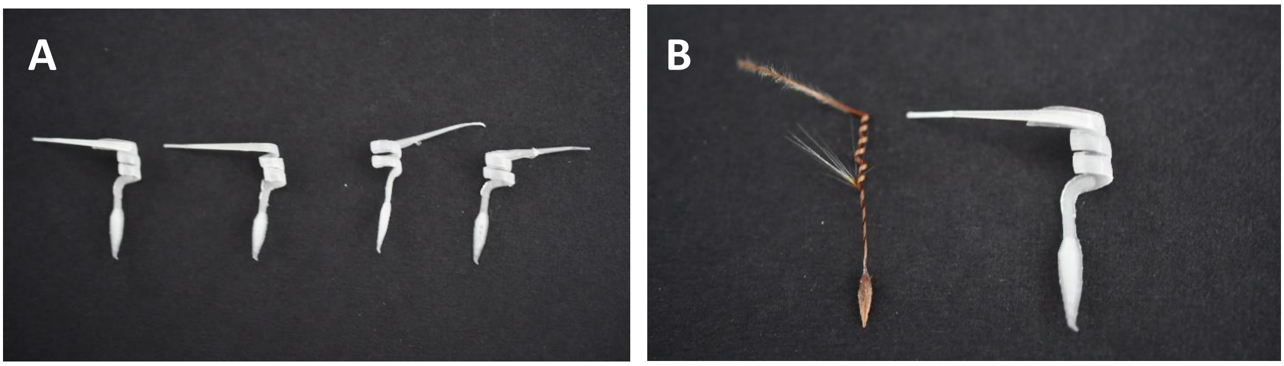

Fig. S21. Examples of samples produced. a) Optical visualization of four different artificial seed. b) Geometrical comparison between natural and artificial seed.

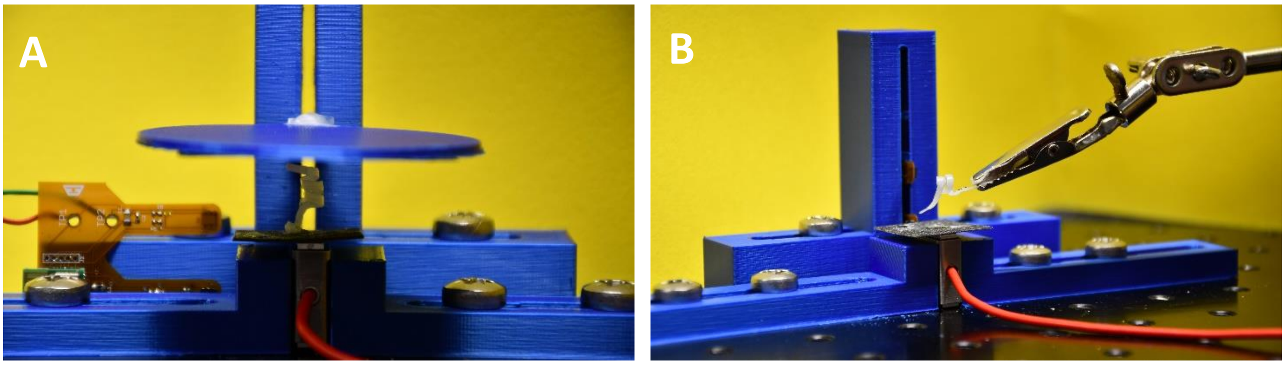

Fig. S22. Experimental setup for extensional force and moment measurements in artificial seed. a) Extensional force measurement. b) Torque measurement

A

B

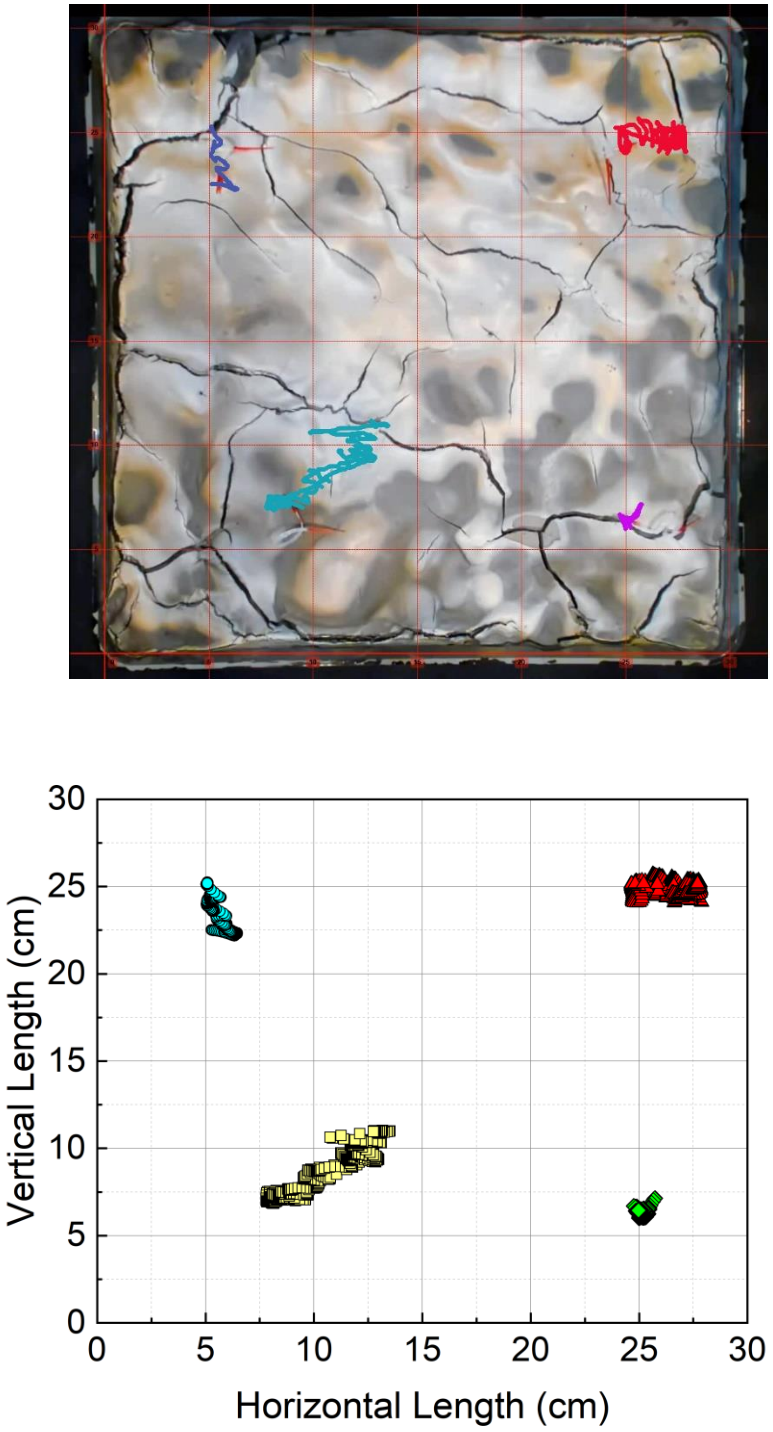

Fig. S23. Soil exploration tracking of artificial seed's capsule. a) Motion tracking provided elaborating Movie S5 with Kinovea software. b) Motion tracking representation in 2D Cartesian coordinate system.

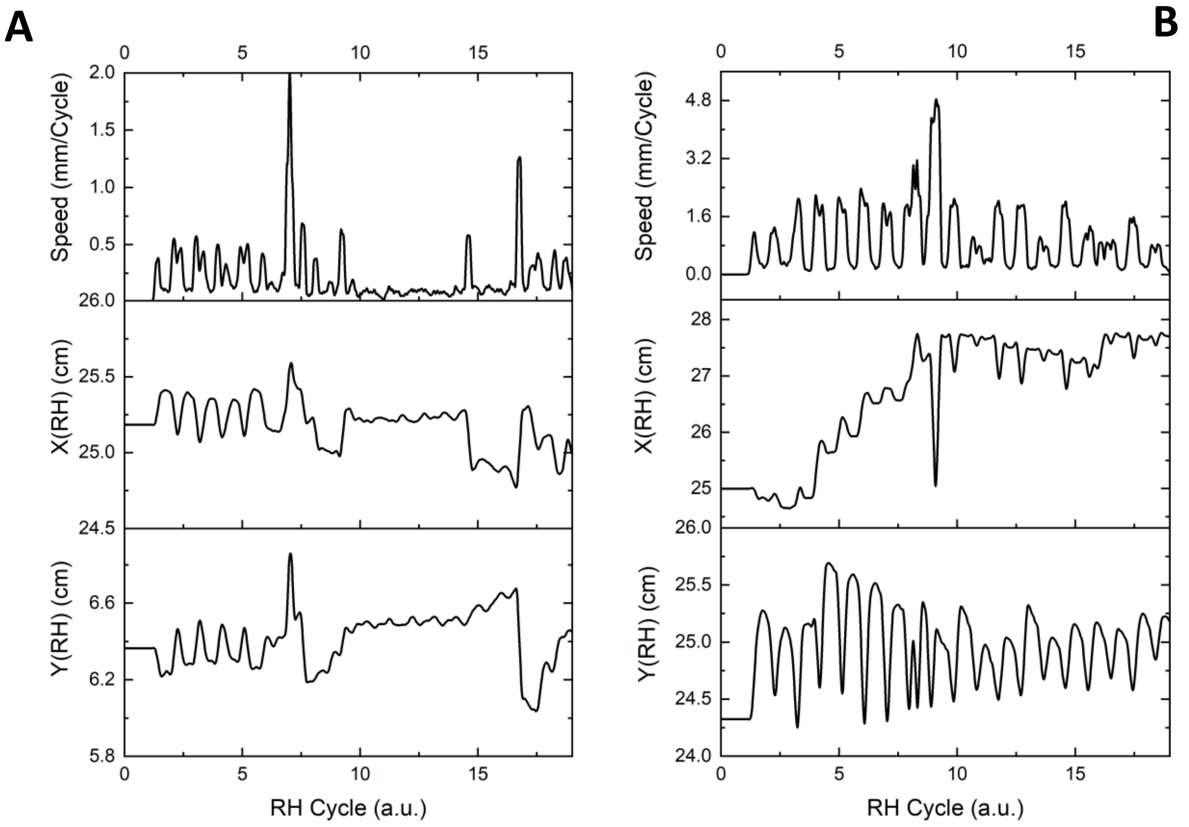

Fig. S24. Experimental evaluation of absolute speed and relative position based on motion tracking. a) South-East artificial seed behaviour. b) North-East artificial seed behaviour.

	Natural Seed		Artificial Seed	
	**Ridge Layer**	**Cap Layer**	**Passive Layer**	**Active Layer**
**Geometrical parameters**				
Length (mm)	18.54±3.1		30.3±1.1	
Width (μm)	428±38		1523±54	
Thickness (μm)	59.66±9.82	39.77±6.86	100.75 ± 4.62	72.93 ± 6.47
Tilt Angle (°)	15±5 (2,6)		15±3	
MFAH (°)	70-80 (2,6)		Equal to Tilt Angle	
**Material Properties**				
Young's Modulus (GPa)	0.780±0.286	1.478±0.249	0.49±0.039	0.254±0.016
Poisson's coefficient	0.33 (7)	0.33 (7)	0.442 (8)	0.36 (9)
CHE (%strain/%RH)	0.06 ± 0.02 (10)	0.20 ± 0.04 (10)	0	0.1 ± 0.03 (2,11)
**Dynamic parameters**				
Weight (mg)	5.2±0.2		21.6±0.4 (12.6±0.6)	
Diffusion Constant (m^2/s)	$2.34 \pm 0.70 \times 10^{-11}$		$4.69 \pm 0.48 \times 10^{-11}$	
Maximum Force (mN)	5.06±1.06		2.40±0.58	
Model Force (mN)	5.65		2.03	
Maximum Torque (μN m)	20.66±2.51		30.42±5.06	
Model Torque (μN m)	57.70		33.35	

Table S1. Summary of geometrical, material, mechanical data and results involved in extrapolation of bioinspired elements for design of artificial seed.

References and Notes

1. Hamann, T., Smets, E., Lens, F. (2011). A comparison of paraffin and resin-based techniques used in bark anatomy. TAXON 60 (3), 841–851, (2011), DOI:10.1002/tax.603016.
2. Jonghyun Ha et al., Hygroresponsive coiling of seed awns and soft actuators, Extreme Mechanics Letters 38 (2020).
3. Wahl A. M., Mechanical springs, Penton Pub. Co., p. 25-222 (1944).
4. Shigley, Joseph E, Charles R. Mischke, and Thomas H. Brown. *Standard Handbook* of *Machine Design*, Chapter 24, p.19, New York: McGraw-Hill, 1996. ISBN 0-07-056958-4.
5. Morrill, E.E., Tulepbergenov, A.N., Stender, C.J., Lamichhane, R., Brown, R.J., Lujan, T.J. A validated software application to measure fiber organization in soft tissue. Biomech Model Mechanobiol 15, 6 (2016).
6. Abraham, Y. and Elbaum, R. (2013), Hygroscopic movements in Geraniaceae: the structural variations that are responsible for coiling or bending. New Phytol, 199: 584-594. https://doi.org/10.1111/nph.12254.
7. Dennis Evangelista, Scott Hotton, Jacques Dumais; The mechanics of explosive dispersal and self-burial in the seeds of the filaree, *Erodium cicutarium* (Geraniaceae). *J Exp Biol* 15 February 2011; 214 (4): 521–529. doi: https://doi.org/10.1242/jeb.050567.
8. Lu, Lin & Zhang, Qingwei & Wootton, David & Chiou, Richard & Lu, Bingheng & Lelkes, Peter. (2014). Mechanical Study of Polycaprolactone-hydroxyapatite Porous Scaffolds Created by Porogen-based Solid Freeform Fabrication Method. Journal of applied biomaterials & functional materials. 12. 10.5301/JABFM.5000163.
9. D.W. Van Krevelen, K. Te Nijenhuis, Properties of Polymers (Fourth Edition), Elsevier, 2009, ISBN 9780080548197, https://doi.org/10.1016/B978-0-08-054819-7.00033-9.
10. Dawson, C., Vincent, J. & Rocca, AM. How pine cones open. Nature 390, 668 (1997). https://doi.org/10.1038/37745
11. Lunni, D., Cianchetti, M., Filippeschi, C., Sinibaldi, E., Mazzolai, B., Plant-Inspired Soft Bistable Structures Based on Hygroscopic Electrospun Nanofibers. Adv. Mater. Interfaces 2020, 7, 1901310
